# Dual RNA-seq analysis reveals differences in defensive lncRNA expression in *Pinus* spp. with varying susceptibility to *Fusarium circinatum*

**DOI:** 10.1186/s12870-026-08182-w

**Published:** 2026-01-23

**Authors:** Cristina Zamora-Ballesteros, Julio J. Diez, Gloria Pinto, Artur Alves, Katrin Heer, Jorge Martín-García

**Affiliations:** 1 Eva Mayr-Stihl Professorship for Forest Genetics, Faculty of Environment and Natural Resources, Albert-Ludwigs-Universität Freiburg, Bertoldstraße 17, 79098 Freiburg i. Br, Germany; 2 Sustainable Forest Management Research Institute iuFOR, University of Valladolid, Avda de Madrid s/n, 34004 Palencia, Spain; 3 Department of Vegetal Production and Forest Resources, University of Valladolid, Avda de Madrid s/n, 34004 Palencia, Spain; 4 Centre for Environmental and Marine Studies, Department of Biology, CESAM, University of Aveiro, Aveiro, 3810-193 Portugal

**Keywords:** Dual RNA-seq, Conifer immunity, *Pinus pinea*, *Pinus radiata*, *Fusarium circinatum*, LncRNA, Co-expression networks

## Abstract

**Background:**

Long non-coding RNAs (lncRNAs) are emerging regulators of plant immunity, but their roles in conifer-pathogen interactions remain largely unexplored. We applied a dual RNA-seq approach to resistant *Pinus pinea* and susceptible *P. radiata* challenged with *Fusarium circinatum* at 4 dpi, and concurrently profiled fungal lncRNAs.

**Results:**

Using a conservative multi-tool pipeline, we identified 8,783 lncRNAs in *P. radiata*, 5,255 in *P. pinea*, and 1,020 in *F. circinatum*. Pine lncRNAs displayed canonical features (shorter length, fewer/shorter exons, intergenic dominance) and limited primary-sequence conservation. Differential expression analysis revealed 37 (*P. radiata*) and 34 (*P. pinea*) infection-responsive lncRNAs. Predicted *cis* targets in *P. radiata* were enriched for energy/redox and gibberellin-related functions, whereas *P. pinea* targets pointed to TCA/redox and translation control. Weighted gene co-expression analysis placed *P. radiata* lncRNAs in defence-like modules without significant infection association, while *P. pinea* lncRNAs clustered into two modules with opposite associations to infection: one that increased and was enriched for immunity, flavonoid biosynthesis, and cell-wall processes, and another that decreased and was linked to photosynthesis and chloroplast functions. On the pathogen side, infection of the resistant host triggered distinct *F. circinatum* lncRNAs linked to transcriptional control and putative *cis* activation of cell-wall–degrading and toxin-biosynthetic genes, alongside down-regulation near ergosterol-biosynthetic loci. By contrast, during infection of the susceptible host, species-associated modules were dominated by translation-related functions.

**Conclusions:**

Together, these results define a non-coding regulatory layer that differentiates resistance and susceptibility strategies in the *Pinus*–*F. circinatum* pathosystem, provide the first lncRNA catalogues for *P. pinea* and *F. circinatum*, and deliver testable candidates for functional validation and biomarker development in forest disease management.

**Supplementary Information:**

The online version contains supplementary material available at 10.1186/s12870-026-08182-w.

## Background

In recent years, our understanding of the molecular mechanisms governing plant responses to pathogenic challenges has expanded significantly. Long non-coding RNAs (lncRNAs; >200 nt) have emerged as key regulatory molecules that fine-tune gene expression at transcriptional and post-transcriptional levels [1]. LncRNAs can be categorized by genomic location into long intergenic ncRNAs (lincRNAs), long intronic ncRNAs (incRNAs) and natural antisense transcripts (NATs) [2]. These transcripts have diverse molecular mechanisms by interacting with different biomolecules such as DNA, RNA and proteins, for example as decoys, guides, enhancers or scaffolds [3, 4]. With the advent of next-generation sequencing and high-resolution transcriptome analyses, plant lncRNA discovery has rapidly advanced in recent years [5]. Several pathogen-responsive lncRNAs have been experimentally validated, including ELENA1 in *Arabidopsis thaliana* [6], lncRNAs induced by *Fusarium oxysporum* [7], and cotton lncNATs whose silencing increased resistance to *Verticillium dahliae* and *Botrytis cinerea* [8]. These studies support a role for lncRNAs in plant immunity, however, lncRNAs remain poorly characterized in non-model plants, particularly in conifer species.

Although transcriptomic studies of interactions in important pathosystems involving conifers have increased considerably over the past decade [9–20], very few have studied the non-coding part [21]. The development of resistant genotypes through breeding and/or genetic engineering requires a deep understanding of the molecular mechanisms underlying plant-pathogen interactions. Using resistant plant material for reforestation may be one of the most effective long-term strategies for mitigating pathogen infections in natural forests and plantations. This holds particularly true when dealing with pathogens that cannot be eradicated, as is the case with *Fusarium circinatum*, the causal agent of Pine Pitch Canker (PPC) [22, 23]. PPC is a destructive disease affecting pines (*Pinus* spp.) and Douglas-fir (*Pseudotsuga menziesii*) globally, causing serious problems in nurseries, pine plantations and natural forests [24]. In this context, various transcriptome analyses aimed at unraveling defence responses have yielded detailed insights into the molecular mechanisms driving disease progression in the *Pinus*-*F. circinatum* pathosystem. These studies have explored host responses, ranging from high susceptibility in pine species like *Pinus greggii*, *P. patula* and *P. radiata*, to moderate or lower susceptibility in *P. maximinoi*,* P. oocarpa*,* P. pinaster*, *P. pinea* and *P. tecunumanii* [11, 13, 17, 18, 20, 25, 26]. The importance of jasmonic acid (JA), ethylene (ET), and salicylic acid (SA) signaling pathways in host resistance to *F. circinatum* has been linked to both resistant and moderately susceptible species. The biosynthesis of secondary metabolites appears to play a significant role in effective defence against the pathogen, with the induction of phenylalanine ammonia-lyase (PAL) expression and higher expression of genes related to this pathway observed in resistant pine species [17, 20]. Additionally, the ineffective response observed in susceptible pine species can likely be attributed to impaired perception of fungal infection and a subsequent failure in downstream defence signaling, as genes encoding pattern recognition receptors (PRRs) and resistance (R) proteins were weakly expressed in these plants [17, 20, 27].

Despite growing evidence that *F. circinatum* infection reprograms host coding and non-coding transcriptomes, including pathogen-responsive lncRNAs linked to kinase signaling, phytohormone regulation, and cell-wall reinforcement [21], the roles of lncRNAs in conifers, and particularly in *P. pinea*, remain unclear. Dual RNA-seq is particularly valuable in this system because it enables simultaneous profiling of host defence and pathogen infection programs from the same infected tissues and time point. Here we use dual RNA-seq to identify and compare defence-related lncRNAs in resistant *P. pinea* and susceptible *P. radiata*, and to profile *F. circinatum* lncRNAs expressed during pathogenesis. We catalog the number and features of host and pathogen lncRNAs, predict *cis* targets, and integrate differential expression with co-expression networks to place lncRNAs within defence modules. Functional annotation of lncRNA targets highlights candidate pathways that may underpin pine resistance and fungal virulence. To our knowledge, this is the first report of *P. pinea* and *F. circinatum* lncRNAs, providing a resource and testable hypotheses for lncRNA-mediated regulation of resistance.

## Methods

### Data collection and reads quality check

Two transcriptome datasets generated from stem tissues of *P. radiata* and *P. pinea* infected with *F. circinatum* [20] were used to identify defence-related lncRNAs. As described in the original study, plant material consisted of one-year-old seedlings from provenance seed lots (Galicia for *P. radiata* and Meseta Norte for *P. pinea*). Each biological replicate corresponded to an individual seedling, and seedlings were randomly allocated to inoculated and mock-inoculated treatments. Seedlings from both species were inoculated with 10 µL of a spore suspension (10^6^ spores mL^− 1^) of the *F. circinatum* isolate Fc072v, originally isolated in northern Spain (Cantabria) and previously described as virulent in *P. radiata* [20, 28, 29].

Control seedlings were mock-inoculated with sterilised distilled water. Plants were maintained in a growth chamber at 21.5 °C under a 16/8 h light/dark photoperiod, watered following routine nursery practice, and no fertilizers or fungicides were applied. Seedlings were kept under these controlled conditions prior to inoculation to allow acclimation.

Sampling at 4 days post-inoculation (dpi) captured the early infection phase (within the first week post-inoculation), when *F. circinatum* biomass increases rapidly [30], and ensures sufficient pathogen biomass for dual RNA-seq analysis. The dataset for each pine species included 4 inoculated and 4 control libraries of strand-specific RNA-seq that were sequenced on an Illumina NovaSeq 6000 platform, and is deposited in Zenodo (doi: 10.5281/zenodo.17591267).

### Plant and pathogen transcript reconstruction

The bioinformatics workflow and versioned scripts are provided in the GitHub repository archived at Zenodo [31]. Fastq files were first assessed for quality control using FastQC v.0.11.9 [32]. The raw reads were trimmed for Illumina adaptor sequences and light 5′-end cropping using Trimmomatic v.0.38 [33]. Quality-filtered reads were then mapped to the *Pinus taeda* reference genome (Pita_v2.01; Treegenes database [34]) using HISAT2 v.2.0.0 [35] with parameters “--dta” and “--rna-strandness RF”. The SAM files from the pine mapping were processed with the SAMtools utility [36] for converting to binary alignment map (BAM) format and sorting by coordinates. The transcripts for each sample were reconstructed separately by StringTie v.2.1.4 [37] using the “-G" option with the annotation file of *P. taeda* (Pita.2_01.entap_annotations.tsv; Treegenes database [34]). This file was previously fixed with Gffread utility v.0.12.1 [38] for the correct readability by StringTie program. After transcripts assembly, 16 GTF files had been generated and subsequently merged based on the pine species using the StringTie “-merge” option. Transcripts with expression levels < 0.1 FPKM (Fragments Per Kilobase of exon per Million) mapped reads were excluded to reduce very lowly supported transcript models, which are more likely to represent spurious reconstructions [39]. Then, two non-redundant set of transcripts (*P. radiata* and *P. pinea*) with unique identifiers were generated and further compared with the *P. taeda* reference annotation GTF file (Pita_v2.01; Treegenes database [34]) using the software GffCompare v.0.12.1 [38]. Transcripts were classified in different class codes according to their nature/origin.

For the pathogen, the identical read alignment and file manipulation process was executed using solely the libraries from the inoculated samples and the sequenced *F. circinatum* Fc072v genome (accession number JAGGEA000000000). As there was no curated GTF file available for this pathogenic fungus, transcript reconstruction was carried out without the aid of an annotation file. Ultimately, the resulting eight GTF files were merged to create a non-redundant set of *F. circinatum* transcripts with unique identifiers, following the same procedure as with the pine files. The transcripts were then annotated using the program Eukaryotic Non-Model Transcriptome Annotation Pipeline (EnTAP) v.0.9.2 [40], which predicts open reading frames (ORFs), conduct a similarity search using DIAMOND v.1.9.2 [41] and assigns protein domains (Pfam), Gene Ontology (GO) terms and KEGG pathways using EggNOG v.1.0.3 [42]. A new GTF file was generated using the annotated transcripts to facilitate comparison with the previous non-redundant set of *F. circinatum* transcripts, categorizing them into different class codes. The annotated transcripts were then discarded from the pipeline (79.3%), and the remaining (20.7%) were retained for lncRNA identification.

### LncRNA identification

Based on all the pine assembled transcripts, the already known transcripts marked with the class code “=” were excluded from each pine transcript set before conducting the potential long non-coding RNAs identification. For the unknown transcripts, stringent conditions were applied using the filter module of the FEELnc v.0.2 tool [43] by removing transcripts < 200 bp and single-exon models to reduce low-confidence short-read assemblies and prioritize a high-confidence lncRNA set. After that, the sequences of the resulting pine transcripts were extracted with Gffread v.0.12.1 [38] and further screened for their respective coding potential using six different computational approaches: [1] Coding Potential Calculator (CPC2 v.1.0.1) [2], Coding-Non-Coding Index (CNCI) [3], Coding-Potential Assessment Tool (CPAT) [4], PLEK [5], FEELnc codpot module and [6] EnTAP v.0.9.2. CPC2 is a tool for assessing protein-coding potential using sequence alignment and biological characteristics, categorizing transcripts as non-coding when the score is < 0 [44]. CNCI analysis distinguishes transcripts by evaluating adjacent nucleotide triplets, not relying on known annotations, where a transcript is considered as non-coding RNA when the score is < 0 [45]. We used a custom CPAT model trained on *F. circinatum* (hexamer/logit), classifying transcripts as non-coding at coding probability < 0.38 [46]. PLEK applies a support vector machine (SVM) algorithm based on an improved k-mer scheme to differentiate lncRNAs from mRNAs [47]. The codpot module of the FEELnc tool for assessing the coding potential of the transcripts was used with the shuffling mode to calculate a coding potential score (CPS) using a random forest algorithm trained by extracting features of ORF coverage, codon usage and nucleotide frequency. The specificity threshold was set at 0.95 to minimize false positives in a non-model, repeat-rich conifer–fungus system. This stringent setting prioritizes precision over recall so that only the most confidently non-coding transcripts are retained. Finally, EnTAP was employed to detect potential annotations among the transcript sequences. In this case, the similarity search was conducted using the NCBI non-redundant protein database (release-201) with E-value < 10^− 5^ as cut-off for identification, excluding genomic databases containing non-coding sequences. For each pine species, candidates for lncRNA transcripts that were consistently identified as non-coding across all methods were accepted as putative lncRNAs.

The lncRNA set for each pine species was further classified into the different GffCompare categories according to the locations relative to the nearest protein-coding genes, including lncRNAs without any overlap with other protein-coding genes are classified as intergenic lncRNAs (lincRNAs; class code ‘u’), natural antisense lncRNAs overlapping exons of a protein-coding transcript on the opposite strand (lncNAT; class code ‘x’), and intronic transcripts (class code ‘i’; [48]).

### Putative characteristics of identified LncRNA transcripts

To understand the differences between lncRNAs and mRNAs, the genomic features of the predicted lncRNAs were analysed focusing on the length, the number of exons and their length, the expression levels and the GC content. For the pine hosts, feature distributions were compared across transcript classes (mRNA, lincRNA, intronic lncRNA and lncNAT) using Kruskal-Wallis tests, followed by Benjamini-Hochberg-corrected pairwise Wilcoxon rank-sum tests. For *F. circinatum*, differences between lncRNA and mRNA transcripts were evaluated using Wilcoxon rank-sum tests with Benjamini-Hochberg correction across features.

The conservation of pine lncRNAs was assessed using two recently updated databases of known plant lncRNAs. CANTATAdb 2.0 [49] includes 239,631 predicted lncRNAs from 36 plant species, while GreeNC 2.0 database [50] encompasses 496,903 lncRNAs from 94 plant and algae species. All the pine transcripts designated as lncRNA were aligned against the known plant lncRNAs using the blastn algorithm (E-value < 10^− 5^) of the BLAST v.2.5.0 software suite. Likewise, the transcripts were also aligned to the Rfam (version 14.10 Nov 2023, 4170 families) and miRBase (version 22.1) non-coding RNA databases using the blastn algorithm (E-value < 10^− 5^) in order to detect housekeeping non-coding RNAs including transfer RNA (tRNAs), ribosomal RNA (rRNAs) and snoRNAs, and miRNA precursors [51, 52].

To explore potential conserved or divergent lncRNAs between both pine species during the fungal infection, we performed a cross-species sequence comparison of the identified lncRNAs. Sequences of lncRNAs from each species were aligned using the blastn algorithm (E-value < 10^− 5^) of the BLAST v.2.5.0 software suite.

### Differential expression analyses between inoculated and control pine seedlings

StringTie was employed to estimate expression for all transcripts of the experiment-level transcriptomes [53]. The output files were reformatted using the “prepDE.py” script for further expression analysis [37]. DESeq2 v.1.40.2 [54] was used to identify differentially expressed lncRNA (DELncRNA) transcripts based on the matrix of the estimated counts. The pairwise comparison of inoculated and control plants for each pine species were evaluated using Wald tests. LncRNAs were considered as differentially expressed if the adjusted p-values for multiple testing, using Benjamini–Hochberg to estimate the false discovery rate (FDR) [55], was less than 0.05 and the |log_2_ (Fold Change)| ≥ 1. To conduct co-expression analysis and assess potential target genes, we identified differentially expressed genes (DEGs) in both species equally.

### LncRNAs target gene prediction and functional enrichment

Based on the genome location of the lncRNAs relative to the neighbouring genes, putative *cis* targets were defined as protein-coding genes located within 10 kb upstream and 100 kb downstream of each lncRNA, following common practice in lncRNA identification pipelines [56]. These genes both in *P. radiata* and *P. pinea* were identified using the FEELnc classifier module [43] and annotated using the EnTAP pipeline [40] as described above but implemented with two additional datasets, the RefSeq complete protein database (release-201) and the UniProtKB/Swissprot database (release-2020_05). Because libraries were strand-specific and read alignment was performed in a strand-aware manner, the assembled transcripts retained strand orientation. Strand and overlap relationships relative to annotated genes (intergenic, antisense exonic overlap, intronic overlap, and divergent/convergent configurations) were derived from GffCompare class codes and FEELnc output, and were used to distinguish antisense-overlapping lncRNAs (lncNATs) from intergenic lncRNAs when interpreting proximity-based *cis* candidates. Functional enrichment analysis of the target genes associated with the DELncRNAs was performed. Utilizing all assembled transcripts of each pine species as background, GO enrichment analysis was conducted using GOSeq v.1.38.0 [57]. This method is based on the Wallenius non-central hyper-geometric distribution, enabling adjustment for transcript length bias. The GO terms with corrected p-values (FDR) lower than 0.05 were considered to be enriched in the group.

### Weighted gene co-expression network analysis (WGCNA) and identification of hub genes

In order to examine the co-expression patterns of protein coding genes and lncRNA genes under infection, read counts matrix for each organism was processed using the Weighted Gene Co-Expression Network Analysis (WGCNA) R package (version 1.73) [58]. This algorithm was used to construct a weighted correlation network and identify sets of highly correlated transcripts (modules) sharing similar expression patterns across samples. Transcripts with fewer than 10 reads and not detected in at least one condition were removed. For the remaining, variance-stabilizing transformation (VST) from DESeq2 was applied prior to network construction. The soft-thresholding power was selected using the scale-free topology criterion (R² ≥ 0.8) while also considering the corresponding slope and mean connectivity to avoid overly sparse networks. Based on this balance, the power was set to 6 for *P. radiata* (R² = 0.952; slope = − 1.88; mean.k = 2,684) and 12 for *P. pinea* (R² = 0.824; slope = − 1.39; mean.k = 852). A signed Topological Overlap Matrix (TOM) was used to quantify network interconnectedness, and modules were defined using the dynamic tree-cut algorithm with a minimum module size of 50 genes and merge cut height = 0.25. Other parameters were kept at default values. Module eigengenes were correlated with the infection trait to identify infection-responsive modules, and modules showing |r| ≥ 0.6 and *p* < 0.05 were considered significantly associated with infection. Within infection-associated modules, the intra-modular connectivity (kME) was examined to identify highly connected genes and lncRNAs (hub candidates). To gain functional insight, GO enrichment analysis of the protein-coding members of each significant module was performed using GOSeq as described above.

## Results

### High-throughput sequencing and transcripts assembly

High-throughput strand-specific RNA-seq of 16 libraries constructed from stem tissue of *P. radiata* and *P. pinea* inoculated with *F. circinatum* and mock-inoculated were analysed for lncRNA identification. Overall mapping rates from both pines and pathogen data sets are provided in Table S1. Sixteen high-depth transcriptomes were generated, with eight derived from each pine species. Within each pine species, four transcriptomes were reconstructed from seedlings inoculated with *F. circinatum*, while the remaining four were obtained from mock-inoculated seedlings. The number of assembled transcripts shared between treatments in each pine species is represented in Venn diagrams (Figure S1). After merging all the transcriptomes separately from each species, the unique transcriptome assembled for *P. radiata* was composed of 90,505 loci and 133,379 transcripts, with 43.1% GC content (Table S2). In the case of the *P. pinea* unique transcriptome, 107,395 transcripts were assembled located in 81,214 loci with 43.8% GC (Table S3). A total of 51,264 (38.44%) transcripts from *P. radiata* and 51,382 (47.84%) transcripts from *P. pinea* matched the *P. taeda* reference annotation (Pita_v2.01.gtf) and were excluded from lncRNA detection. These transcripts correspond to annotated protein-coding RNAs. The remaining 82,110 (*P. radiata*) and 56,013 (*P. pinea*) transcripts were processed through the lncRNA identification pipeline.

In the case of *F. circinatum*, the transcripts were assembled in a similar manner. Eight transcriptomes were generated, with four reconstructed from *P. radiata* seedlings and four from *P. pinea* seedlings, all of them inoculated with the pathogen. After merging all transcriptomes, the unique non-redundant transcriptome assembled for the fungus was composed of 10,486 loci and 11,706 transcripts, with 50.3% GC content (Table S4). The EnTAP program annotated 9,282 transcripts (79.3%), and the remaining 2,424 (20.7%) were utilized in the lncRNA identification pipeline (Table S5).

### Genome-wide identification and characterization of pine LncRNAs

**Fig. 1:**
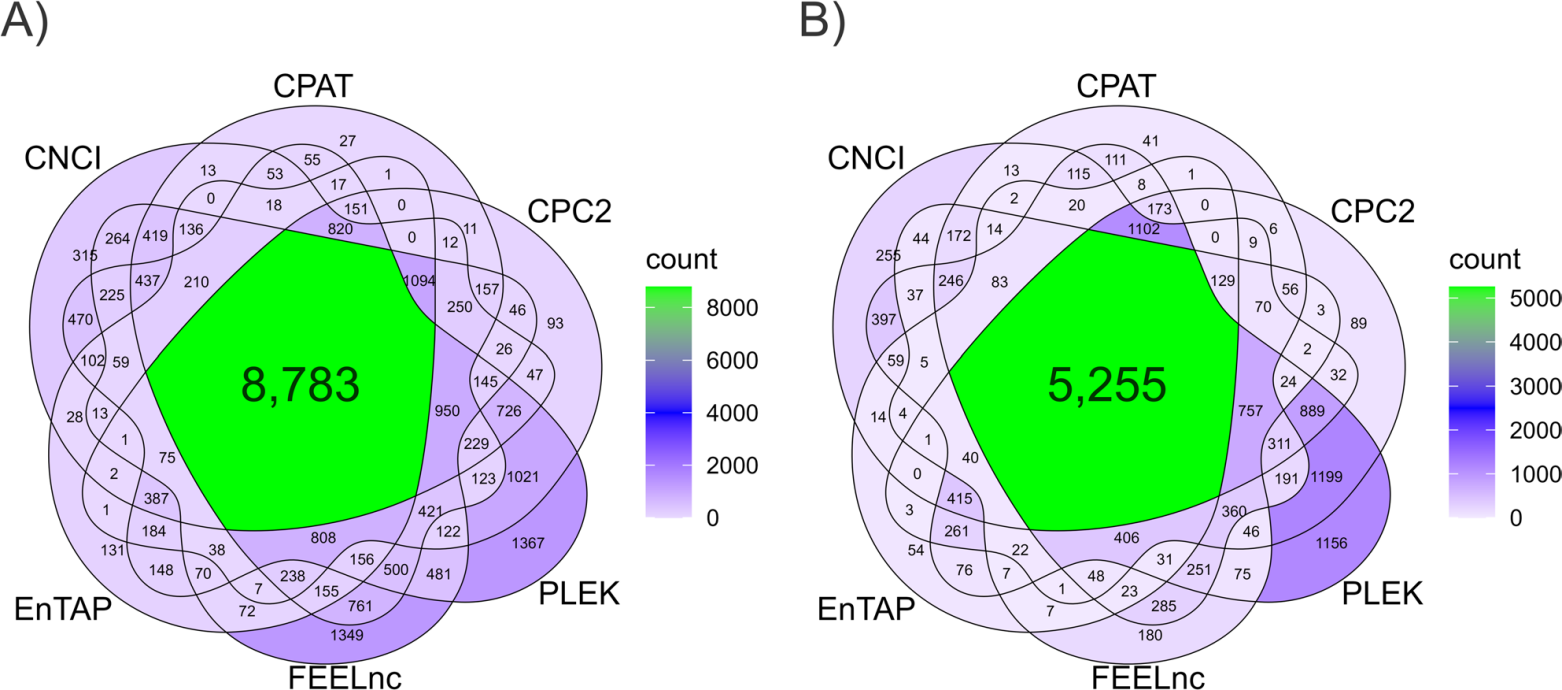
Venn diagrams showing the numbers of candidate lncRNAs in (**A**) *P. radiata* and (**B**) *P. pinea* according to the Coding Potential Calculator (CPC2), Coding-Non-Coding Index (CNCI), Coding-Potential Assessment Tool (CPAT), PLEK, FEELnc codpot module and the Eukaryotic Non-Model Transcriptome Annotation Pipeline (EnTAP)

Unknown transcripts from each pine species were subjected to sequential filtering to obtain candidate lncRNAs. First, FEELnc excluded 54,597 (66.5%) and 38,964 (69.6%) transcripts in *P. radiata* and *P. pinea*, respectively, based on length and structure. We then assessed coding potential using six approaches (CPC2, CNCI, CPAT, PLEK, FEELnc and EnTAP). PLEK yielded the highest number of non-coding candidates, whereas EnTAP yielded the lowest, likely reflecting its primary role in annotation rather than coding-potential prediction. After removing candidates with coding potential, we retained 8,783 (*P. radiata*) and 5,255 (*P. pinea*) lncRNAs (Fig. 1). These lncRNAs were further categorized into different class codes based on their relationship with the closest reference transcripts. Table 1 summarizes the most representative class codes of assembled transcripts and predicted lncRNAs for each species. Percentages refer to the proportion within the total number of assembled transcripts and within the total number of predicted lncRNAs.

**Table 1:** Classification of *P. radiata* and *P. pinea* transcripts according to gffcompare class codes

Class code	After assembly		LncRNAs predicted		Description^1^
	Pinus radiata Transcript no.	Pinus pinea Transcript no.	Pinus radiata Transcript no.	Pinus pinea Transcript no.	
**x**	550 (0.41%)	303 (0.28%)	142 (1.62%)	72 (1.37%)	Natural antisense overlapping an exon of an annotated gene at the opposite strand (lncNATs)
**i**	1,199 (0.90%)	964 (0.90%)	218 (2.50%)	163 (3.10%)	Fully contained in a known intron (intronic lncRNAs)
**y**	559 (0.42%)	331 (0.31%)	160 (1.82%)	129 (2.46%)	Contains a reference gene within its intron
**u**	49,678 (37.25%)	34,635 (32.25%)	8,262 (94.10%)	4,889 (93.04%)	Intergenic region (lincRNAs)

^1^Brief explanation of the class codes

**Fig. 2:**
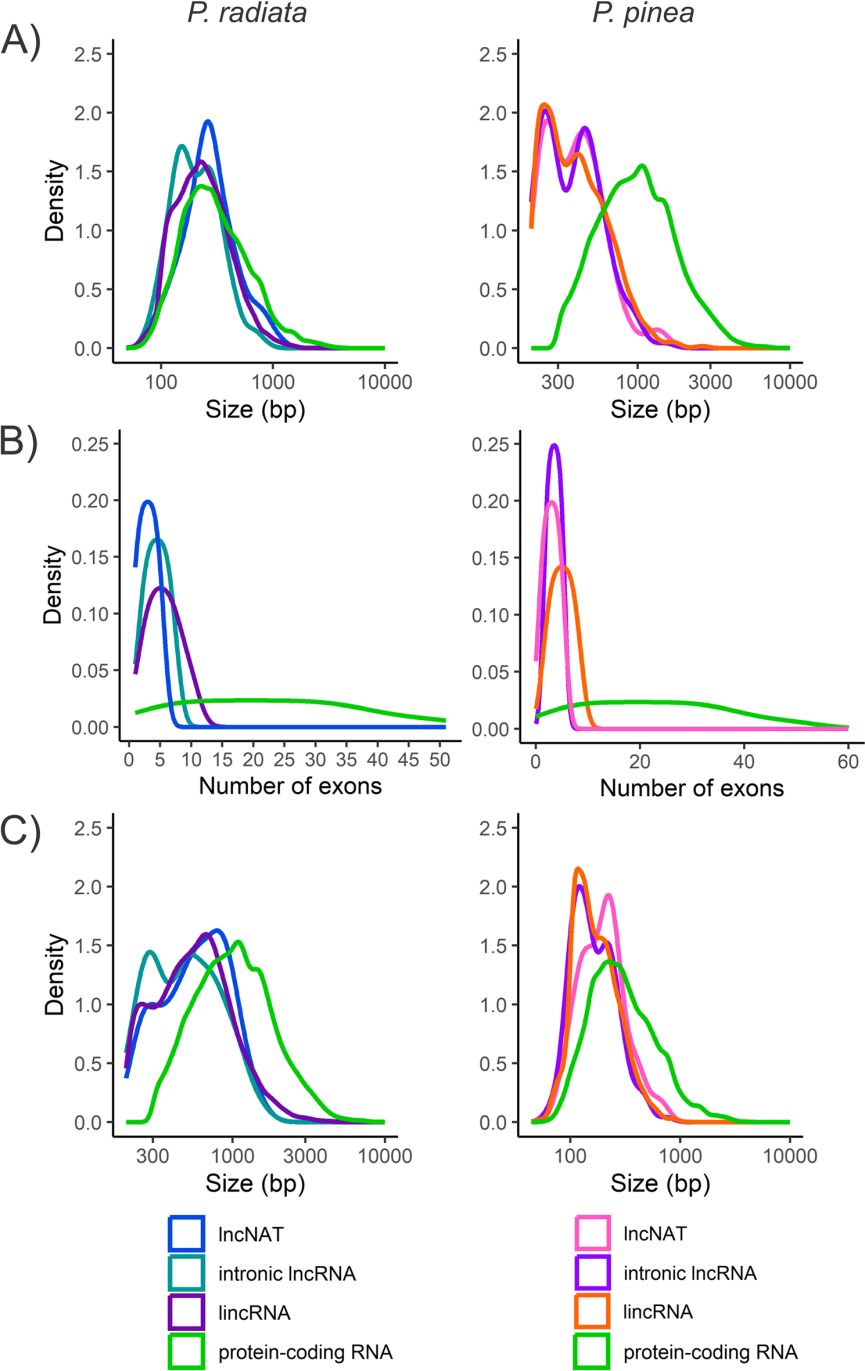
Characterization of pine lncRNA transcripts showed differences with the characteristics of protein-coding transcripts in *P. radiata* and *P. pinea*. (**A**) Transcript size distribution, (**B**) number of exons per transcript, and (**C**) exon size distributions.

**Fig. 3:**
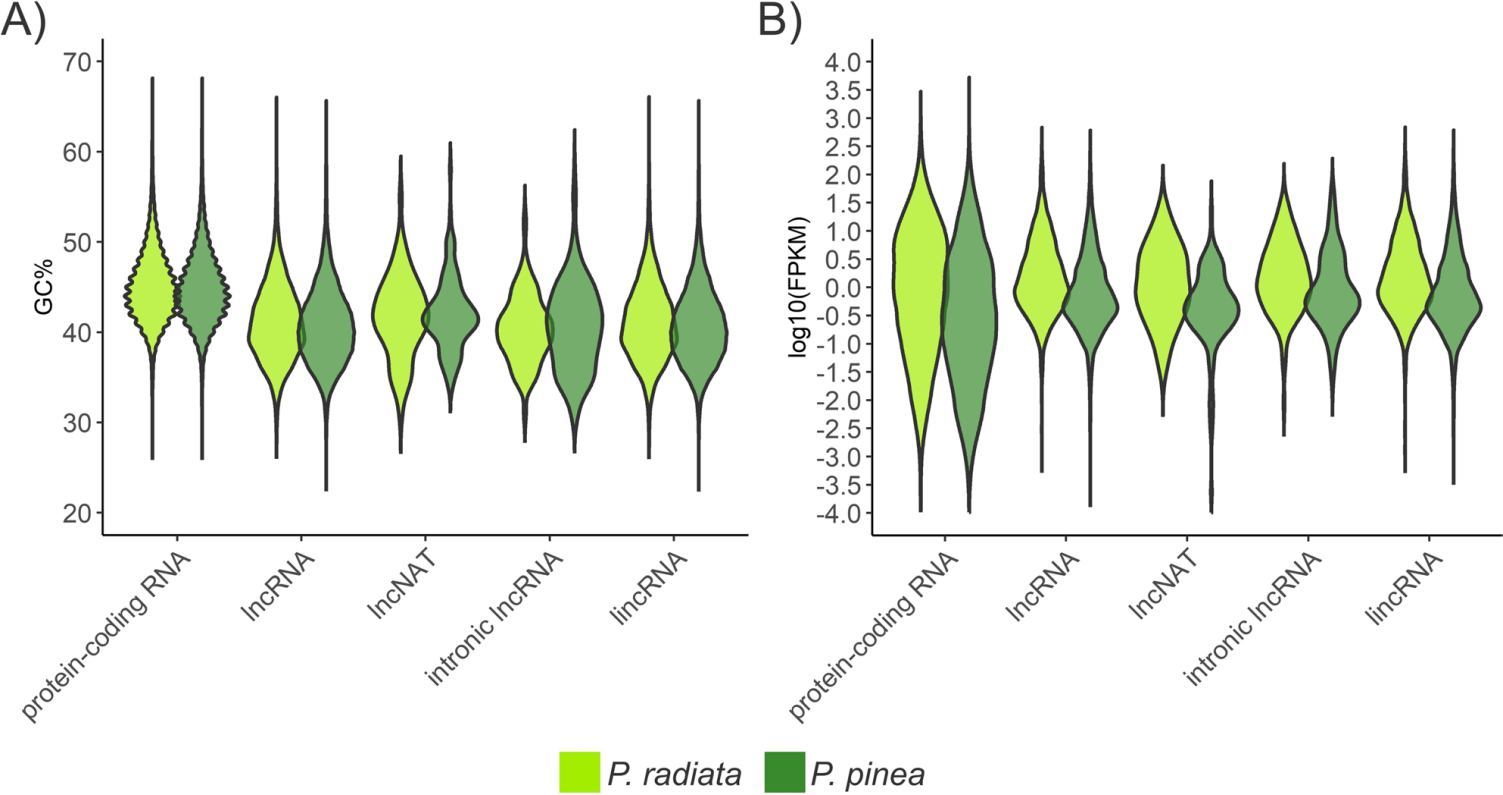
Differences in (**A**) GC content and (**B**) Fragments Per Kilobase of exon per Million (FPKM) distribution for lncRNAs and protein-coding RNAs in *P. radiata* and *P. pinea*.

The results revealed that the majority of lncRNA transcripts were categorized as lincRNAs in both pine species, other classes being much less represented (Table 1). Differences in the characteristics of lncRNA transcripts compared to those of the protein-coding transcripts were identified in both species (Figs. 2 and 3) and were supported by non-parametric tests across transcript classes (Table S6). The average length of protein-coding transcripts of *P. radiata* (1,205 bp) and *P. pinea* (1,169 bp) was higher than that of lincRNAs (653 and 442 bp), lncNATs (632 and 417 bp) and intronic lncRNAs (557 and 411 bp). In general, lengths of *P. radiata* lncRNAs (614 bp) appeared to be longer than those of *P. pinea* (428 bp), while differences among lncRNA subclasses were generally smaller. The number of exons per transcript was significantly higher in the protein-coding transcripts than in the lncRNA transcripts in both species. The average exon length was 394 bp and 386 bp in protein-coding, and 272 bp and 192 bp in lncRNA transcripts of *P. radiata* and *P. pinea*, respectively. The GC content of pine lncRNAs (41%) was lower than that of protein-coding transcripts (45%) (Fig. 3A), whereas GC differences among lncRNA subclasses were modest, with lncNATs showing slightly higher values. Likewise, lncRNAs showed lower expression than mRNAs (2.6 vs. 5.0 FPKM in *P. radiata*; 5.4 vs. 9.4 FPKM in *P. pinea*), and overall expression differences among lncRNA subclasses were limited. Notably, *P. radiata* lncNATs differed from other lncRNA classes, with slightly higher GC content (42% vs. ~40.6%) and lower expression (1 FPKM vs. ~3.1 FPKM). The same tendency was observed in *P. pinea* lncNATs, although less pronounced.

We identified 82 lncRNAs in *P. radiata* and 19 in *P. pinea* as putative miRNA precursors (Tables S7-S8). These candidates belonged to 15 miRNA families, with MIR160 and MIR1314 most represented in *P. radiata*, and MIR160 and MIR3701 in *P. pinea*. In addition, Rfam annotated 69 transcripts in *P. radiata* and 35 in *P. pinea* (Table S9), spanning 13 conserved RNA families (rRNAs, tRNAs, histones, and snoRNAs). Notably, no histones or snoRNAs were detected among *P. pinea* transcripts.

**Fig. 4:**
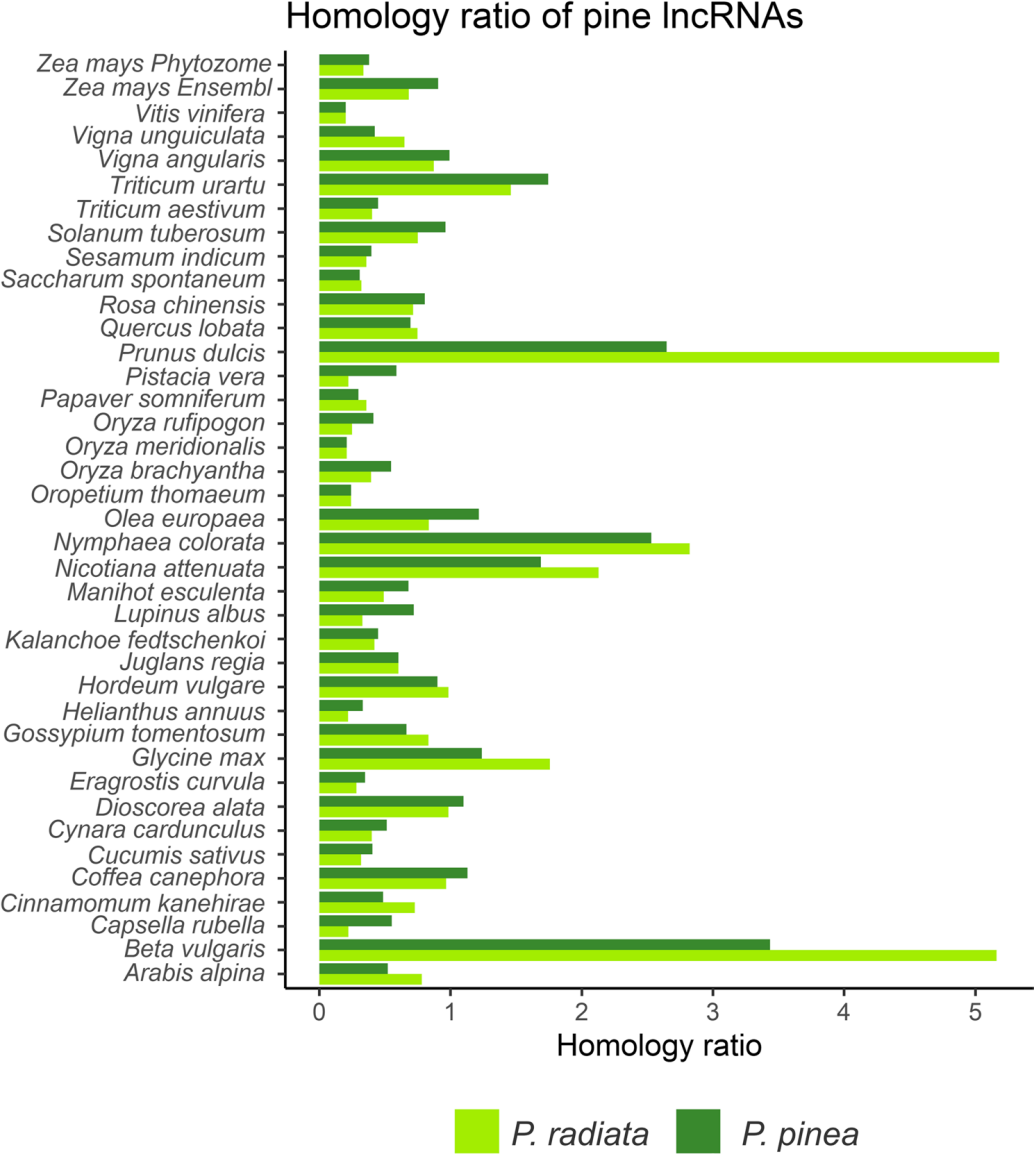
Homology ratios of lncRNAs from *Pinus radiata *and *P. pinea* to known lncRNAs in the CANTATA 2.0 and GreeNC 2.0 databases. Bars represent the number of hits for each plant species relative to its total lncRNAs. Only ratios > 0.2 are shown.

To assess conservation at the sequence level, we searched for homologous lncRNAs in public datasets. Pine lncRNAs were aligned against 493,512 annotated lncRNAs from 94 plant species in GreeNC 2.0 (Table S10) and against the CANTATA 2.0 dataset (Table S11). Notably, these resources do not include gymnosperms. To avoid redundancy, we selected species so they did not appear in both databases. Overall, 1,991 (22.7%) lncRNAs of *P. radiata* and 2,057 (39.1%) lncRNAs of *P. pinea* exhibited homology with at least one hit with a known lncRNA from other plant species. Both pine species exhibited similar homology ratios with known plant lncRNAs (Fig. 4). The highest ratios, determined by the number of hits of pine lncRNAs relative to the total number of lncRNAs for each plant species, were consistently observed with *Beta vulgaris* (3.4% in *P. radiata* and 5.2% in *P. pinea*) and the woody plant *Prunus dulcis* (2.7% in *P. radiata* and 5.2% in *P. pinea*) in both pine species.

### Genome-wide identification and characterization of pathogen LncRNAs

**Fig. 5:**
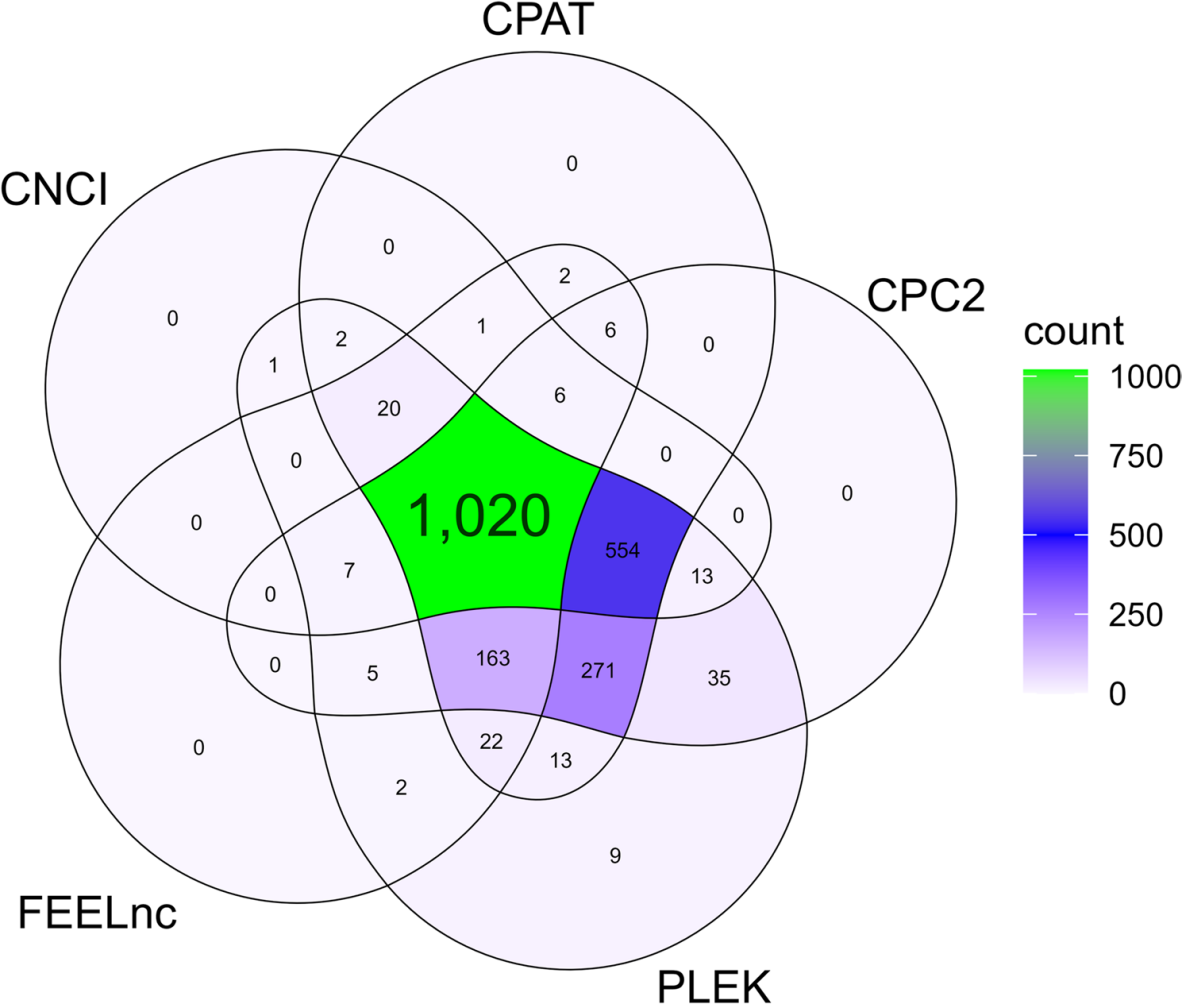
Venn diagrams showing the numbers of candidate lncRNAs in *F. circinatum* according to Coding Potential Calculator (CPC2), Coding-Non-Coding Index (CNCI), Coding-Potential Assessment Tool (CPAT), PLEK and FEELnc codpot module.

The fungal transcripts that were not annotated using the EnTAP program (2,424) were subjected to sequential filtering steps to obtain the lncRNA transcripts. Based on transcript length and structure, the FEELnc filter module excluded 267 transcripts (11.01%) of *F. circinatum*. Next, the protein-coding potential of transcripts was predicted using five computational approaches (CPC2/CNCI/CPAT/PLEK/FEELnc). PLEK identified the highest number of non-coding transcripts (2,137 predicted lncRNAs), with the FEELnc tool being the most stringent, predicting 1,254 lncRNAs. A total of 1,020 transcripts were predicted as lncRNAs for the pathogen consistently across all approaches (Fig. 5). After categorizing the lncRNAs into different class codes, the majority of the lncRNAs were lincRNAs with 935 (91.7%) transcripts, followed by lncNAT with 75 (7.4%) transcripts, nine transcripts with the intron matching on the opposite strand, and one intronic transcript. In addition, one lncRNA was identified as miRNA precursor (MIR3250) using miRBase database (Table S7), and two others were annotated as rRNAs and tRNAs using Rfam database (Table S9).

**Fig. 6:**
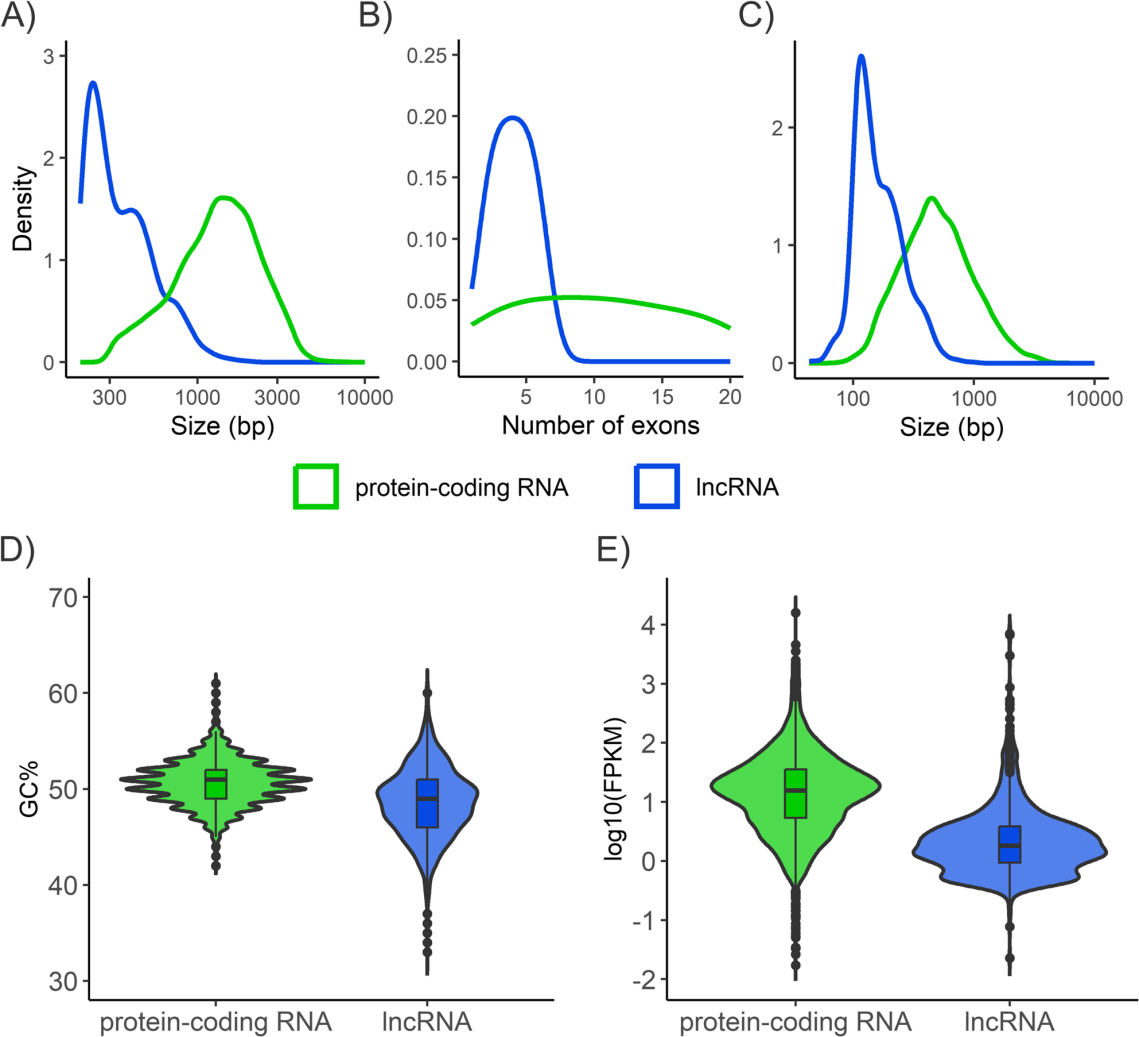
Characterization of lncRNA transcripts showed differences with the characteristics of protein-coding transcripts in *F. circinatum*. **A** Transcript size distribution, (**B**) number of exons per transcript, (**C**) exon size distributions, (**D**) GC content and (**E**) FPKM distribution for lncRNAs and protein-coding RNAs.

Overall, pathogen lncRNAs were considerably shorter (average = 388 bp, mode = 206 bp) than protein-coding transcripts (average = 1,519 bp, mode = 730 bp; Fig. 6A), and this difference was statistically supported (Table S6). Differences in the analysis of the exons were also observed. LncRNAs had fewer exons (median = 2) than protein-coding transcripts (median = 3; *p* < 0.001; Fig. 6B), with two-exon lncRNAs being the most frequent class. Consistent with transcript length, lncRNA exons were shorter (average = 181 bp, mode = 137 bp) than mRNA exons (average = 626 bp, mode = 430 bp; *p* < 0.001; Fig. 6C). The GC content of lncRNAs (48.5%) was slightly lower than that of protein-coding transcripts (50.6%; *p* < 0.001; Fig. 6D). Finally, lncRNAs showed lower expression (median log_10_(FPKM) = 0.26 vs. 1.20; *p* < 0.001; Fig. 6E).

### Differential expression analysis in pine-*F. circinatum* interaction

**Fig. 7:**
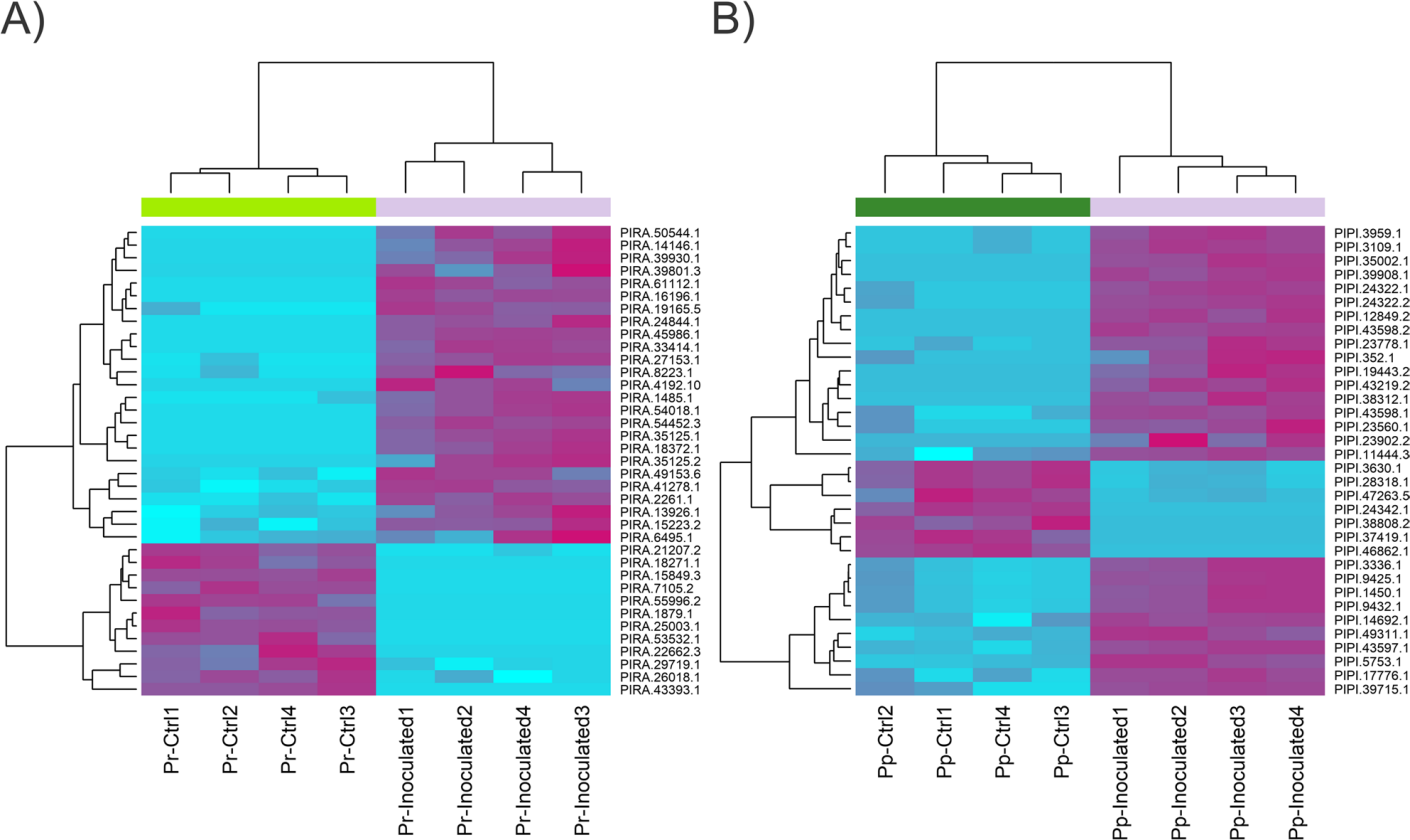
Hierarchical clustering plot of the differentially expressed lncRNAs of (**A**) *P. radiata* and (**B**) *P. pinea* in response to *F. circinatum*. The plot shows the scaled expression levels of the lncRNAs. Different columns represent different libraries, and different rows represent the differentially expressed lncRNAs. Purplish: relatively high expression; Blue: relatively low expression

The expression changes of lncRNAs and protein-coding transcripts between *F. circinatum*-inoculated seedlings and controls were analyzed for the two different pine species separately. The PCAs revealed different patterns for each treatment (control or inoculated, Figure S2). A total of 37 lncRNA transcripts were identified as differentially expressed (adjusted p-value < 0.05, log_2_ (|Fold-change|) ≥ 1) in response to pathogen infection in *P. radiata*, with 25 up-regulated and 12 down-regulated (Fig. 7A). Among the differentially expressed lncRNAs (DELncRNAs), 35 were lincRNA transcripts, while the remaining consisted of one lncNAT and one lncRNA transcript containing a coding-protein in its intron (Table S12). One of the down-regulated DELncRNAs (PIRA.18271.1) was found to be homologous to a snoR64 sequence. In the case of *P. pinea*, 34 lncRNA transcripts were differentially expressed on infection, with 27 up-regulated and 7 down-regulated (Fig. 7B). These lncRNAs were categorized as lincRNAs [31], lncNATs [1] and intronic lncRNAs [2] (Table S13). The volcano plots displaying the top differentially expressed lncRNAs in both species are shown in Figure S3. On the other hand, 812 (618 up-regulated and 194 down-regulated) and 1,037 (830 up-regulated and 207 down-regulated) protein-coding transcripts showed significantly differential expression in *P. radiata* and *P. pinea* (inoculated vs. control), respectively (Table S14 and S15). DEGs were clustered in heat maps and represented in volcano plots in order to visualize the expression pattern of both conditions of the analyses (Figure S4 and S5).

**Fig. 8:**
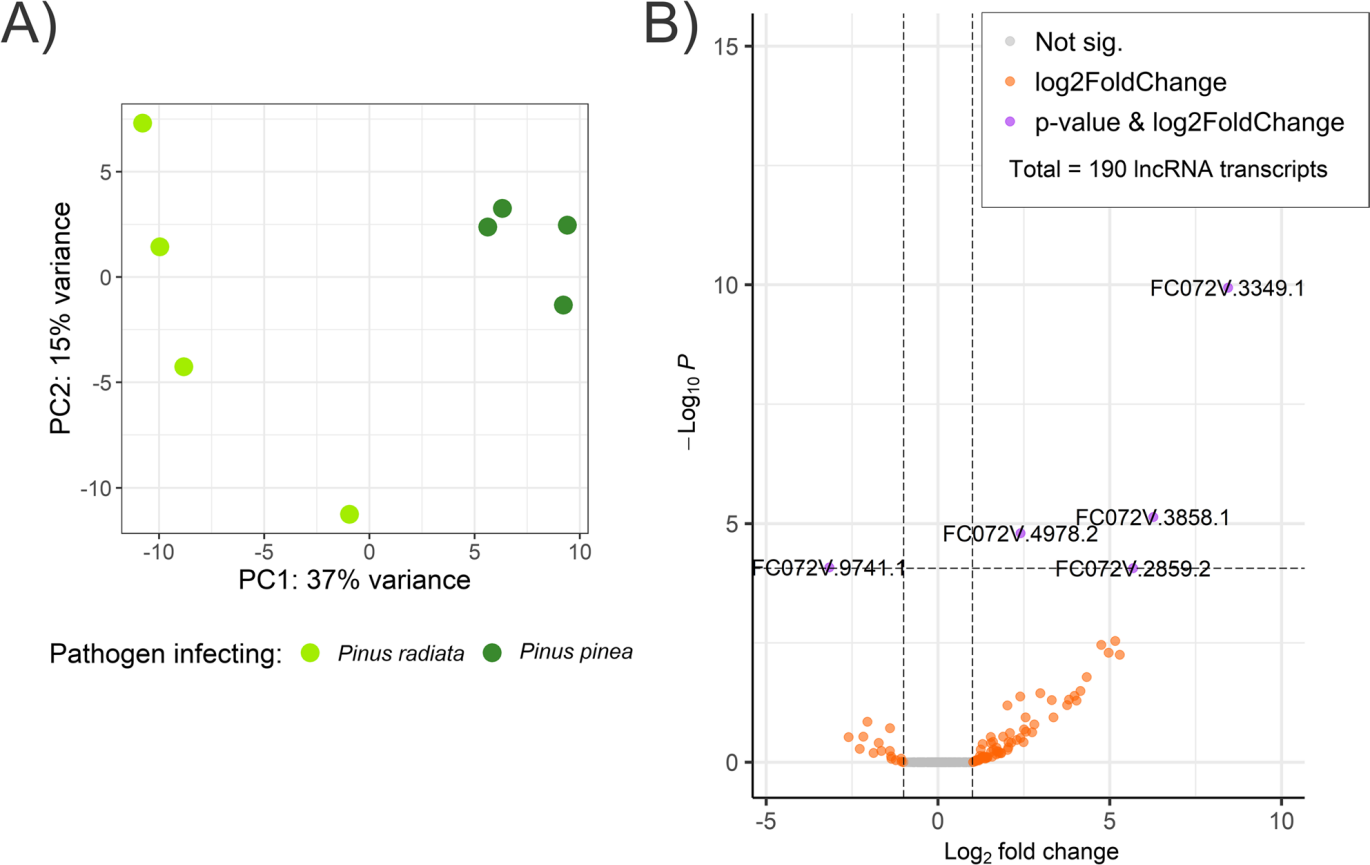
Visualization of differential expression analysis (DEA) of lncRNAs identified in *F. circinatum*. **A** Two-dimensional scatterplot of the principal component analysis (PCA) for pathogen lncRNAs, based on rlog-transformed counts. **B** Volcano plot illustrating the differentially expressed lncRNAs (DELncRNAs) in *F. circinatum* infecting *P. radiata* compared to infecting *P. pinea*. The x-axis represents log2 fold change, and the y-axis represents log10 p-value. Labeled transcripts represent the DELncRNA IDs (adjusted p-value < 0.05 and |log2 fold change| ≥ 1).

PCA revealed different patterns for *F. circinatum* lncRNAs infecting each species (Fig. 8A). The differential expression analysis for the pathogen infecting *P. pinea* compared with infecting *P. radiata* revealed four up-regulated and one down-regulated lncRNAs (Fig. 8.B). It’s worth noting that three of the up-regulated lncRNAs were not expressed by the pathogen during the infection to *P. radiata* (FC072V.3858.1, FC072V.3349.1, FC072V.2859.2). Among the DELncRNAs, four were lincRNA transcripts, and one lncNAT (Table S16). The expression analysis of the protein-coding transcripts of *F. circinatum* revealed 216 DEG, with 145 up-regulated and 71 down-regulated genes in *F. circinatum* infecting *P. pinea* compared to infecting *P. radiata* (Table S17 and Figure S6).

### Analysis of lncRNAs *cis*-interacting genes

**Fig. 9:**
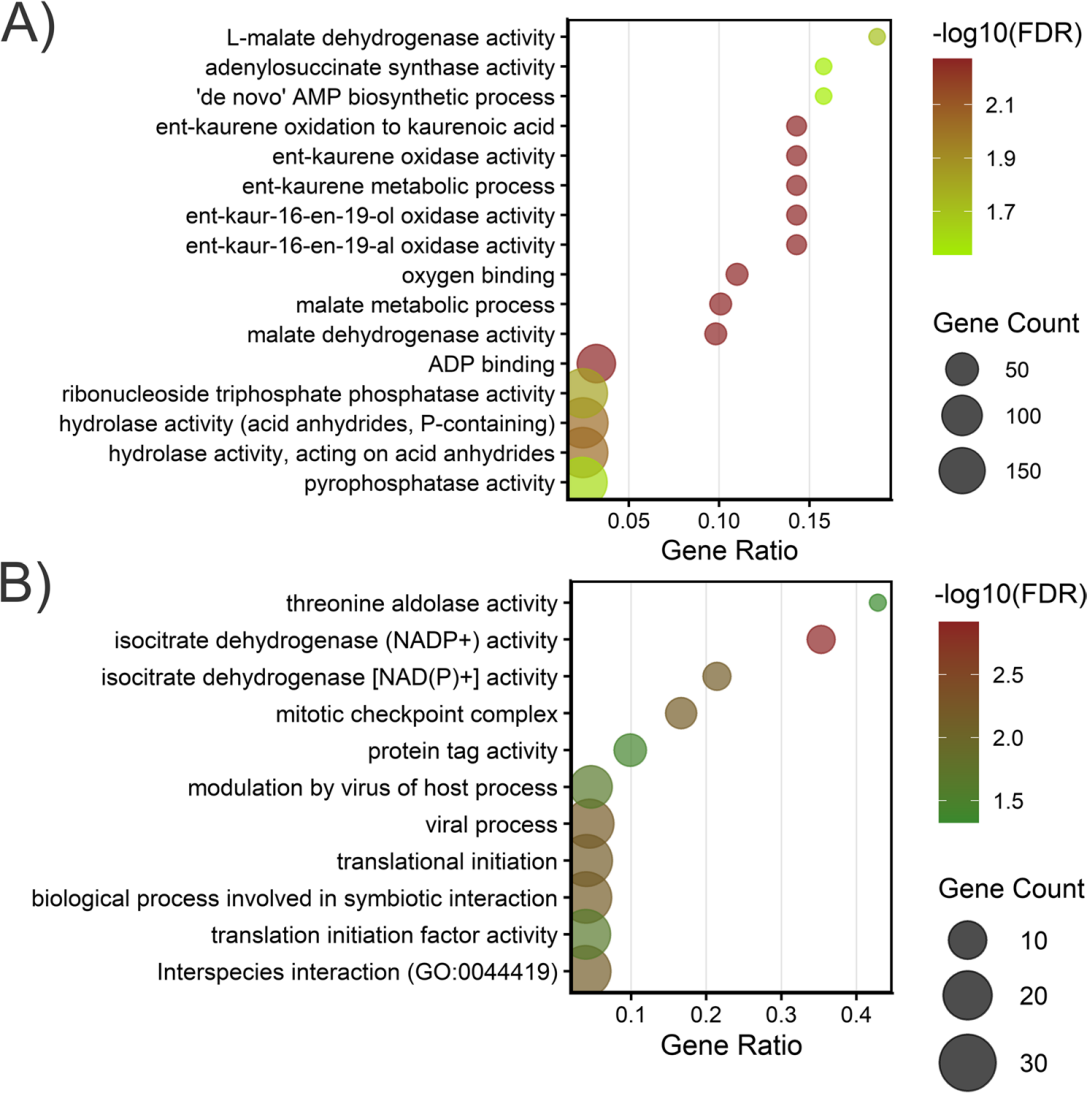
Gene Ontology (GO) enrichment of *cis*-associated target genes predicted for lncRNA–mRNA interaction pairs in *Pinus radiata* (**A**) and *Pinus pinea* (**B**). Only categories with FDR < 0.05. Gene count represents the number of input genes annotated to each GO term. The Gene ratio is Gene count divided by the total number of input genes retained in the enrichment test.

To predict the role of *cis*-acting lncRNAs in response to *F. circinatum* in both pine species, protein-coding transcripts located within a 10 kb window upstream and 100 kb downstream were investigated. A total of 2,577 and 1,892 lncRNA–mRNA interaction pairs were recorded in *P. radiata* and *P. pinea*, respectively, by the FEELnc classifier module. However, one lncRNA could have more than one target gene, and a target gene could be the target of one or more lncRNAs. In *P. radiata*, a number of 1,776 candidate *cis* target genes were observed for 2,267 lncRNAs (Table S18), of which 2,027 had a single candidate target gene and 240 lncRNAs had multiple interactions. The maximum number of target genes for a single lncRNA was five, which was reached by ten lncRNAs (Table S19). Putative *cis* targets were enriched for nucleotide/energy metabolism and gibberellin (GA) biosynthesis, suggesting an involvement in these pathways (Fig. 9A). In *P. pinea*, 1,422 candidate cis-target genes were identified for 1,654 lncRNAs (Table S20), of which 1,480 were associated with a single target gene, while 174 lncRNAs showed multiple interactions. The lncRNA PIPI.27358.5 was predicted to have potential *cis* interactions with eight distinct protein-coding transcripts, while five other lncRNAs exhibited five plausible interactions each with different genes (Table S21). Functional enrichments of the potential targets pointed to TCA/redox, and biotic interface/translation control (Fig. 9B).

Eight DELncRNAs in *P. radiata* displayed potential *cis* interactions with eight distinct candidate target genes. Notably, one of these target genes, predicted to have a phosphofructokinase domain, exhibited up-regulation in a magnitude similar to its potential regulator (Table 2). In contrast, *P. pinea* had eleven DELncRNAs, each with the potential to interact with an equal number of candidate target genes, including one up-regulated in line with its potential DELncRNA regulator (Table 2). The functional prediction for these DELncRNAs was based on the functional annotation of their nearby target genes. Among the targeted genes in *P. radiata*, there were genes involved in molecular signalling encoding protein kinases, genes with domains commonly found in proteins associated with pathogen resistance (TIR, NB-ARC), one gene related to cell-wall reinforcement and lignification (4-coumarate-CoA ligase), and one plant-specific transcription factor (NAC) involved in the regulation of stress responses. In *P. pinea*, DELncRNAs potentially targeted genes involved in cell redox homeostasis, including a gene encoding for a thioredoxin reductase (NTRA), hydrolysis (transcripts with PAP2 domains) and cellular stress (transcript with DnaJ domain), among others. One of these genes predicted to be *cis*-regulated was also associated with brassinosteroid responses, a plant hormone that plays key roles in plant adaptation to biotic stresses.

**Table 2:** Candidate target genes predicted to interact with DELncRNA transcripts

LncRNA	Log2FC^a^	Target gene	Log2FC^a^	Direction and type^b^	Location	Description of targeted gene
*Pinus radiata*						
PIRA.53532.1	6.23 ↓	PITA_11394		Sense, intergenic (125)	Same strand, upstream	Phosphatidylinositol phosphate kinase (PIP5K9)
PIRA.15849.3	7.84 ↓	PITA_42244		Sense, intergenic (13,546)	Same strand, upstream	Transcript with toll/interleukin-1 receptor homology (TIR) domain
PIRA.61112.1	7.37 ↑	PITA_48846		Antisense, intergenic (865)	Divergent, upstream	Transcript with NB-ARC domain
PIRA.41278.1	2.66 ↑	PITA_19394		Antisense, genic	Overlapping, intronic	NAC transcription factor
PIRA.21207.2	7.17 ↓	PITA_10260		Sense, intergenic (46,688)	Same strand, downstream	Calcium/calmodulin dependent protein kinase I
PIRA.26018.1	3.78 ↓	PITA_29813		Antisense, intergenic (80,313)	Convergent, downstream	Probable galactinol-sucrose galactosyltransferase 6 isoform X1
PIRA.24844.1	6.47 ↑	PITA_43179		Sense, intergenic (5,950)	Same strand, downstream	4-coumarate-CoA ligase, partial (4CL3)
PIRA.39930.1	8.48 ↑	PITA_33393	7.87 ↑	Sense, genic	Containing, intronic	Transcript with phosphofructokinase domain
*Pinus pinea*						
PIPI.37419.1	6.60 ↓	PITA_20432		Sense, intergenic (90,655)	Same strand, downstream	RIX1
PIPI.46862.1	5.76 ↓	PITA_14476		Antisense, genic	Containing, intronic	Transcript with phosphofructokinase domain
PIPI.23778.1	4.56 ↑	PITA_41443		Sense, intergenic (5,563)	Same strand, upstream	Unknown
PIPI.47263.5	3.16 ↓	PITA_48741		Sense, intergenic (555)	Same strand, upstream	Transcript with PAP2 domain
PIPI.49311.1	3.50 ↑	PITA_22808		Antisense, genic	Nested, intronic	Carboxyl-terminal-processing peptidase 1, chloroplastic, partial
PIPI.19443.2	7.50 ↑	PITA_02979		Sense, genic	Nested, intronic	Transcript with phosphofructokinase domain
PIPI.38312.1	6.41 ↑	PITA_11059		Sense, intergenic (9,141)	Same strand, upstream	Transcript with DnaJ domain
PIPI.17776.1	3.70 ↑	PITA_09873		Antisense, intergenic (21,628)	Divergent, upstream	Thioredoxin reductase NTRA
PIPI.11444.3	3.57 ↑	PITA_23988		Sense, intergenic (402)	Same strand, downstream	EXORDIUM-like protein
PIPI.38808.2	6.53 ↓	PITA_08782		Sense, intergenic (2,603)	Same strand, downstream	Transcript with phosphofructokinase domain
PIPI.39715.1	6.34 ↑	PITA_36678	9.71 ↑	Sense, intergenic (200)	Same strand, downstream	Transcript with PAP2 domain
*Fusarium circinatum*						
FC072V.9741.1	3.16 ↓	FC072V.9742.1		Sense, intergenic (5069)	Same strand, downstream	ERG24
		FC072V.9739.1		Sense, intergenic (9910)	Same strand, upstream	Integral membrane protein
		FC072V.9743.1		Sense, intergenic (6863)	Same strand, downstream	Homogentisate 1,2-dioxygenase
		FC072V.9744.1		Sense, intergenic (9576)	Same strand, downstream	Hypothetical protein FGLOB1_6208
FC072V.4978.2	2.40 ↑	FC072V.4980.1		Sense, intergenic (1555)	Same strand, downstream	Fusaric acid biosynthesis 9 (FUB9)
		FC072V.4976.2		Sense, intergenic (7495)	Same strand, upstream	Cutinase transcription factor 1 beta (CTF1-BETA)
		FC072V.4977.1		Antisense, intergenic (5028)	Convergent, downstream	Major facilitator superfamily transporter
		FC072V.4981.1		Sense, intergenic (4049)	Same strand, downstream	Linear gramicidin synthase subunit D
		FC072V.4976.1		Sense, intergenic (7495)	Same strand, upstream	Cutinase transcription factor 1 beta
		FC072V.4982.1		Antisense, intergenic (8116)	Divergent, upstream	O-acetylhomoserine (thiol)-lyase
		FC072V.4981.2		Sense, intergenic (4157)	Same strand, downstream	Linear gramicidin synthase subunit D
FC072V.3858.1	6.27 ↑	FC072V.3856.1		Sense, intergenic (2726)	Same strand, upstream	Hypothetical protein FVER53263_00133
		FC072V.3855.1		Sense, intergenic (3573)	Same strand, upstream	Related to hsp70 protein
		FC072V.3854.1		Sense, intergenic (6770)	Same strand, upstream	Rhamnogalacturonan acetylesterase
FC072V.3349.1	8.44 ↑	FC072V.3348.1		Antisense, genic	Overlapping, exonic	Hypothetical protein FBULB1_308
		FC072V.3351.2		Sense, intergenic (5515)	Same strand, downstream	Beta-glucosidase G (BGLG)
		FC072V.3350.1		Sense, intergenic (615)	Same strand, downstream	Related to transcription factor PLM2
		FC072V.3351.1		Sense, intergenic (5317)	Same strand, downstream	Beta-glucosidase G (BGLG)
		FC072V.3346.1		Sense, intergenic (9233)	Same strand, upstream	Transcript with ring finger domain
FC072V.2859.2	5.68 ↑	FC072V.2860.1		Antisense, intergenic (216)	Convergent, downstream	MED7-member of RNA polymerase II transcriptional regulation mediator complex
		FC072V.2853.1		Antisense, intergenic (9383)	Divergent, upstream	Proteasome subunit alpha type-4
		FC072V.2856.1		Sense, intergenic (5406)	Same strand, downstream	CTI6 Cyc8-Tup1 interacting
		FC072V.2857.1		Sense, intergenic (1012)	Same strand, downstream	Adenylosuccinate synthetase (ADSS)
		FC072V.2861.1		Antisense, intergenic (2679)	Convergent, downstream	F-box domain-containing protein

^a^The symbol ↑ refers to up-regulated expression and ↓ refers to down-regulation expression of lncRNAs and genes

^b^Numbers in parenthesis indicate the genomic distance between the lncRNA and its potential target gene

**Fig. 10:**
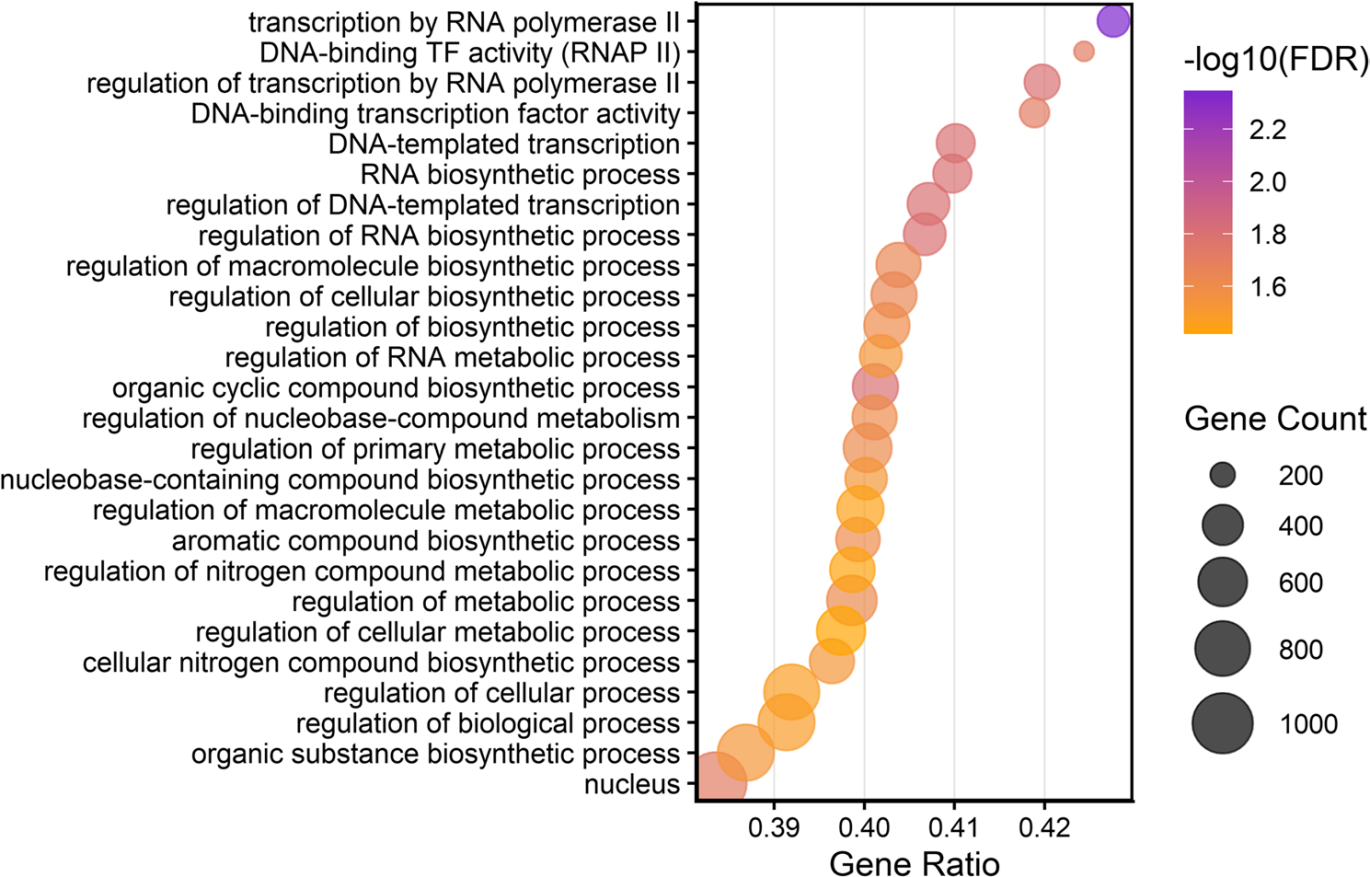
Gene Ontology (GO) enrichment of predicted *cis*-associated target genes for lncRNA–mRNA interaction pairs in *F. circinatum*. Only GO terms with FDR < 0.05. Gene count represents the number of input genes annotated to each GO term. The Gene ratio is Gene count divided by the total number of input genes retained in the enrichment test.

Similarly, the *cis*-acting activity of lncRNAs of *F. circinatum* was based on their location with respect to protein-coding transcripts. The number of lncRNA–mRNA interaction pairs predicted in *F. circinatum* was 4,584 (Table S22). A number of 3,298 candidate *cis* target genes were observed for 984 lncRNAs, of which 920 (87.3%) had multiple interactions. The majority of lncRNAs were predicted to interact with three distinct protein-coding transcripts. The lncRNA FC072V.10163.1 had the highest number of target genes, reaching thirteen (Table S23). Functional enrichment analysis of these candidate targets revealed a strong bias toward transcriptional regulation and biosynthetic processes (Fig. 10). Five DELncRNAs showed potential *cis* interactions with 24 candidate target genes (Table 2), none of which belonged to the set of differentially expressed genes. Specifically, the down-regulated DELncRNA in the pathogen infecting *P. pinea* compared with *P. radiata* (FC072V.9741.1), potentially *cis*-regulates genes associated with ergosterol biosynthesis and phenylalanine degradation. On the other hand, potential target genes of up-regulated DELncRNAs in *F. circinatum* infecting *P. pinea*, were implicated in protein degradation, including genes with F-box and RING finger domains, mycotoxin biosynthesis (fusaric acid), and transcriptional regulation, such as CTF1-BETA and MED7, the latter being involved in the regulated transcription of nearly all RNA polymerase II-dependent genes. It’s worth noting that two potential target genes with hydrolysis activity and only expressed during the infection to *P. pinea*, are associated with the degradation of plant cell walls, including rhamnogalacturonan and cellulose. The target genes regulating the DELncRNAs were not enriched for any GO term.

### Weighted gene co-expression network analysis associated with pine defence response

**Fig. 11:**
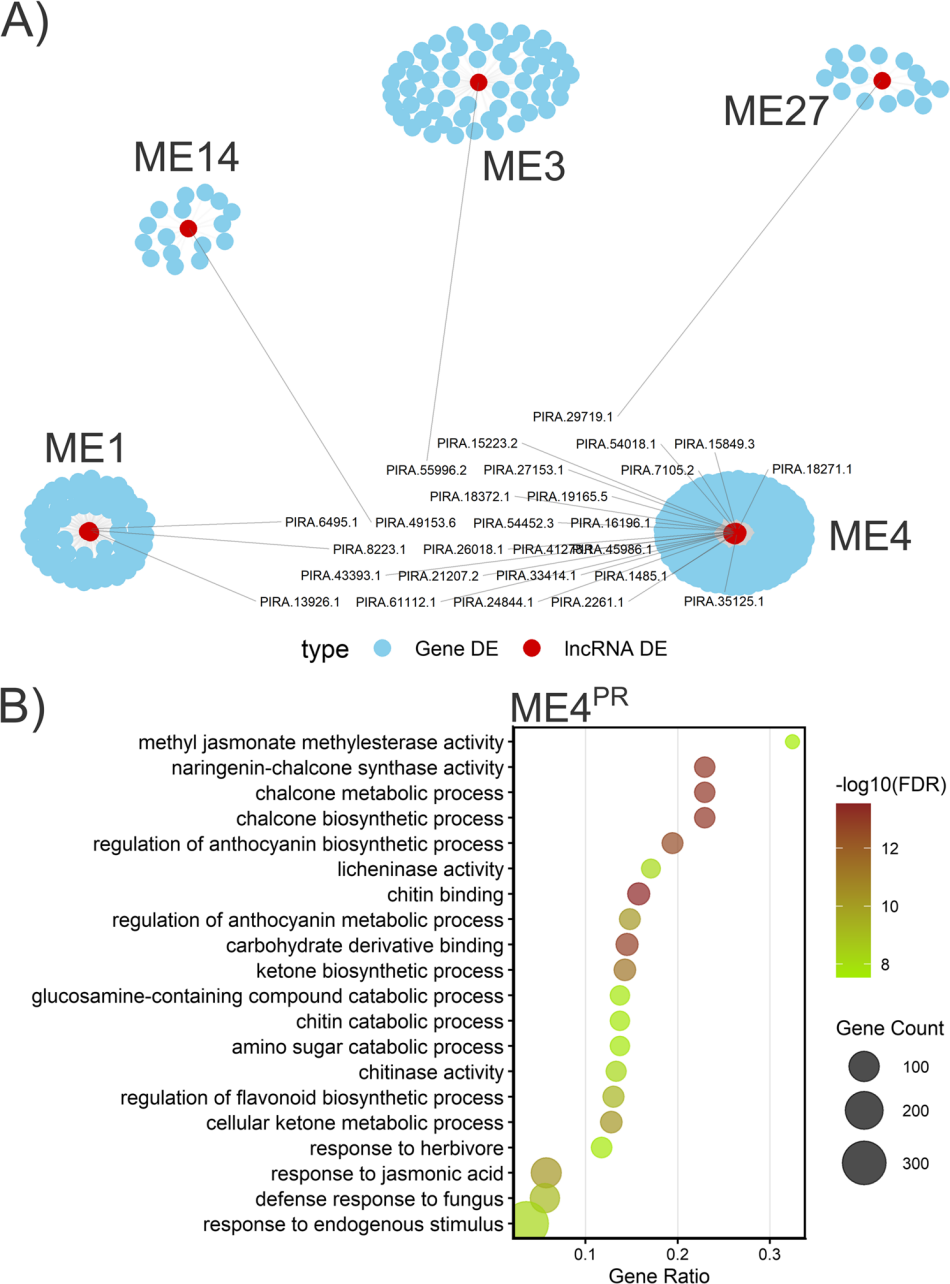
Co-expression modules containing DELncRNAs in *P. radiata*. **A** Global network of DELncRNAs and DEGs in common modules. Red nodes represent DELncRNAs and blue nodes represent DEGs. **B** Top 20 enriched Gene Ontology (GO) biological processes associated with genes from module ME4PR, which harbored the highest number of DELncRNAs. Gene count represents the number of input genes annotated to each GO term. The Gene ratio is Gene count divided by the total number of input genes retained in the enrichment test

A WGCNA was constructed on a filtered transcript dataset consisting of 83,366 and 65,524 transcripts in *P. radiata* and *P. pinea*, respectively, after removing low count reads to study the potential roles of *trans*-regulating lncRNAs in pine defence. Soft-thresholding powers were set to 6 in *P. radiata* and 12 in *P. pinea*, representing a balance between scale-free topology approximation and network connectivity (Figure S7). For *P. radiata*, the analysis identified 72 modules containing similar patterns of expression. Five of these modules (ME1^PR^, ME3^PR^, ME4^PR^, ME14^PR^, ME27^PR^) harboured lncRNAs that were differentially expressed upon *F. circinatum* infection, however, none of these modules showed a significant correlation with the infection condition (Figure S8). These modules were enriched for different biological processes (Table S24; Figure S9). We then built a global co-expression network of these DELncRNAs and DEGs, revealing 688 transcripts interconnected by 10,897 edges across all common modules (Fig. 11). Hub analysis in the largest such module identified ME4^PR^ as having the greatest number of DELncRNAs [21]. This module was therefore selected for further study. Hub connectivity (kME > 0.95) within ME4^PR^ highlighted 11 and 505 highly connected DELncRNAs and DEGs, respectively. The functional enrichment of the coding genes in ME4^PR^ (3,425) returned 178 significantly over-represented GO terms (FDR < 0.05; Table S24). The top categories included general defence response, chitin binding, chalcone metabolic process, chalcone biosynthetic process, and naringenin-chalcone synthase activity (Fig. 11B). These results suggest that the ME4^PR^ lncRNAs may participate in *trans*-regulation of key defence pathways, especially those related to cell-wall modification and flavonoid biosynthesis, during the infection.

**Fig. 12:**
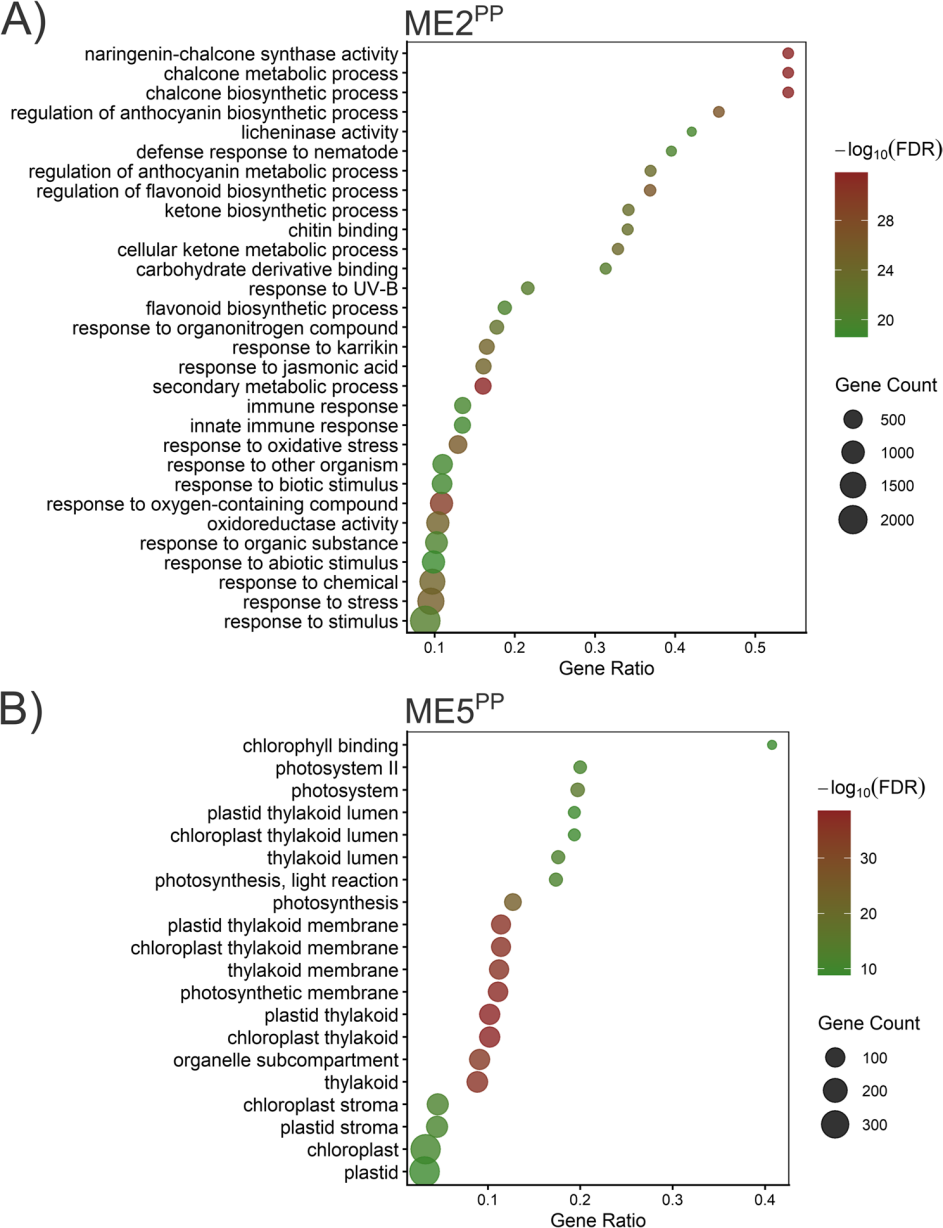
Top enriched Gene Ontology (GO) terms associated with genes from infection-associated modules (**A**) ME2PP (positive correlation with infection, r = 0.885) and (**B**) ME5PP (negative correlation, r = −0.897), harboring DELncRNAs in *P. pinea*. Gene count represents the number of input genes annotated to each GO term. The Gene ratio is Gene count divided by the total number of input genes retained in the enrichment test.

For *P. pinea*, the analysis identified 75 modules containing similar patterns of expression. Five modules were significantly correlated with the infection condition (Figure S10). In this case, DELncRNAs were detected in two of these infection-associated modules, ME2^PP^ showing a strong positive correlation (*r* = 0.885, *p* = 0.0035) and ME5^PP^ a strong negative correlation (*r* = − 0.897, *p* = 0.0025). We then built a global co-expression network of these DELncRNAs and DEGs, revealing 846 transcripts interconnected by 17,241 edges across all common modules (Figure S10). Within ME2^PP^ (8,110 transcripts), hub analysis (kME > 0.95) identified 1,421 hub genes, including 7 DELncRNAs and 735 DEGs. Functional enrichment of the coding genes in ME2^PP^ returned 554 significantly over-represented GO terms (FDR < 0.05; Table S25; Fig. 12A). Within ME5^PP^ (2,444 transcripts), hub analysis identified 121 hub genes (none of them DELncRNAs) and 84 DEGs. The enrichment analysis yielded 116 GO terms (FDR < 0.05; Table S25; Fig. 12B). Together, these results suggest that *P. pinea* DELncRNAs tend to be concentrated in infection-associated modules with opposite signs of association, with ME2^PP^ harboring most of the DELncRNAs and displaying broad enrichment for defence-related functions. In contrast, ME5^PP^ was enriched in photosynthesis- and chloroplast-related processes, suggesting a reprogramming of primary metabolism associated with its negative correlation to infection. Together, *P. pinea* displayed infection-responsive modules enriched for defence-relevant functions, placing DELncRNAs at well-defined network positions. By contrast, *P. radiata* defence-associated modules containing DELncRNAs showed weak or no robust association with infection, consistent with a diffuse transcriptional response.

### Weighted correlation network analysis associated with *F. circinatum* pathogenesis

**Fig. 13:**
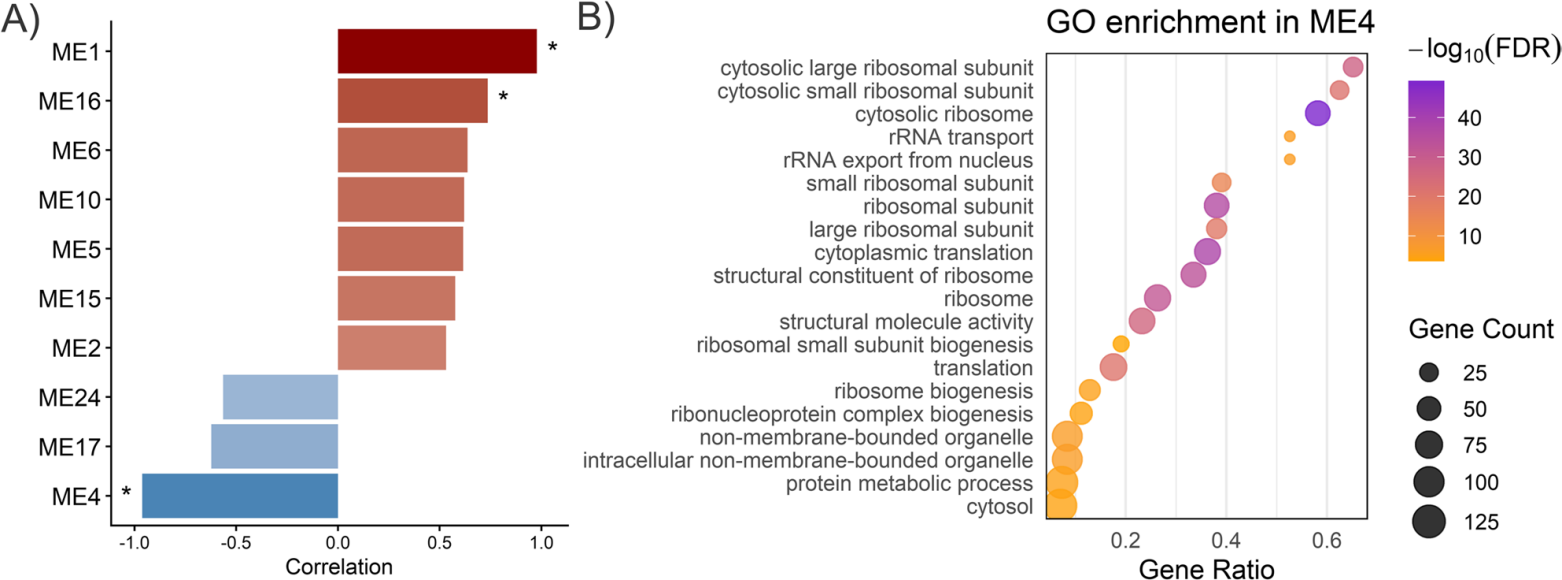
Co-expression modules associated with *F. circinatum* pathogenesis infecting resistant or susceptible pine species. **A** Barplot of the top 10 modules ranked by absolute correlation with host species. Asterisks indicate modules with significant correlations (|r| > 0.6, p < 0.05). **B** Top 20 significantly enriched Gene Ontology (GO) terms for coding genes in module ME4, which was more highly expressed in P. radiata. Gene count represents the number of input genes annotated to each GO term. The Gene ratio is Gene count divided by the total number of input genes retained in the enrichment test

For the pathogen dataset, the co-expression analysis was conducted on 6,787 transcripts after filtering for low expression. The soft-thresholding power was set to 12 (R² = 0.878; slope = − 1.51; mean.k = 85.5), and the adjacency function was used to construct the adjacency matrix (TOMType = signed, minModuleSize = 30, mergeCutHeight = 0.25; Figure S11A). Hierarchical clustering of samples based on variance-stabilized counts showed clear separation according to the host species infected by the pathogen (Figure S11B). A total of 31 co-expression modules were identified, of which three (ME1^FC^, ME16^FC^, and ME4^FC^) were significantly correlated with the host species (Fig. 13A). Modules ME1^FC^ and ME16^FC^ showed positive correlations, being more highly expressed in *P. pinea*, while ME4^FC^ showed a negative correlation and was therefore associated with *P. radiata*. DELncRNAs were detected in ME1^FC^ [4] and ME4^FC^ [1]. In ME1^FC^ (1,457 transcripts), hub analysis (kME > 0.95) identified 55 hub transcripts, including 3 DELncRNAs, alongside 141 DEGs. Functional enrichment of coding genes in this module yielded a single significant category related to the extracellular region (Table S26). In contrast, ME4^FC^ contained 441 transcripts, including 35 DEGs and 70 hub genes, but no DELncRNAs among the hubs. Enrichment analysis of ME4^FC^ highlighted 49 over-represented categories, strongly dominated by ribosome- and translation-related functions as well as RNA binding and processing (Table S26; Fig. 13B). Together, these results suggest that pathogen-derived DELncRNAs tend to localize within species-associated modules.

### Cross-species comparison

Sequence similarity was also assessed between the identified lncRNAs of both species. The analysis revealed that 3,567 lncRNAs were shared between the pine species (percentage identity > 75%; Table S27), representing 40.6% of *P. radiata* lncRNAs and 67.9% of *P. pinea* lncRNAs. Among them, only one common differentially expressed lncRNA was shared between the species (PIRA.54452.3/PIPI.43598.2), highlighting the low level of conservation among lncRNAs involved in the defence response to *F. circinatum* and/or potential species-specific regulatory mechanisms. Notably, the expression of this putative homologous lncRNA was similarly upregulated in both species, with a fold change of 11.8 in *P. radiata* and 9.1 in *P. pinea*. In the co-expression networks, PIRA.54452.3 belonged to ME4^PR^, where it was identified as a hub (kME: 0.957) and the module showed strong enrichment for chalcone/flavonoid biosynthesis, chitin response, and jasmonate signaling. Its homolog PIPI.43598.2 was placed in ME2^PP^, which was also enriched for these defence-related processes, but it did not reach hub status, suggesting conserved functional associations but divergent network centrality.

We identified several lncRNAs that were differentially expressed in *P. pinea* under pathogen infection, while their homologs in *P. radiata* were not (Table S28). These lncRNAs are therefore potentially associated with resistance mechanisms. Conversely, *P. radiata* DELncRNAs whose homologs in *P. pinea* were not involved in the defence response may represent susceptibility-associated regulators. Some of these lncRNAs were predicted to regulate protein-coding genes (Table S28). For instance, a susceptibility-associated lncRNA was predicted to *cis*-regulate a glycosyl hydrolase (PITA_29813), whereas a resistance-associated lncRNA was predicted to influence a thioredoxin reductase (*NTRA*).

We focused on representative DELncRNAs and their cross-species homologs that potentially regulate the expression of the same coding gene, showing functionally conserved nodes (Table 3). DELncRNAs/homologs pairs belonged to co-expression modules (ME2^PP^ and ME4^PR^) strongly enriched for classic defence and specialized-metabolism programs (JA/SA signaling, chitin recognition/catabolism, phenylpropanoid/flavonoid and terpenoid biosynthesis, oxidative-stress and cell-wall remodeling terms). By contrast, *P. radiata* ME3^PR^, which contains the thioredoxin reductase (PITA_09873) targeted by the *P. pinea* DELncRNA PIPI.17776.1, is dominated by translational machinery and mitochondrial respiration functions. These patterns show that lncRNAs converge on conserved defence targets (e.g., PITA_29813, PITA_09873) but act through different co-expression modules.

**Table 3:** DELncRNAs and their homologs in pine species that share the same predicted coding gene targets

DELncRNA	WGCNA module	Homolog	WGCNA module	Predicted target	WGCNA module	Predicted target annotation
PIRA.26018.1	ME4^PR^	PIPI.19356.1	ME1^PP^	PITA_29813	n.d.	Galactinol–sucrose galactosyltransferase; Sip1 / raffinose pathway
PIPI.11444.3	ME2^PP^	PIRA.16474.4	n.d.	PITA_23988	n.d.	EXORDIUM-like protein; growth/abiotic stress, possible wall remodeling
PIPI.17776.1	ME5^PP^	PIRA.24069.1	ME3^PR^	PITA_09873	ME3^PR^ (hub gene, kME = 0.952)	Thioredoxin reductase; redox/ROS

## Discussion

The importance of the non-coding genome in plant biology has become evident over recent decades [59]. In model and crop species, lncRNAs have been shown to act across immunity layers, from perception and signalling to transcriptional and post-transcriptional control. They may operate in *cis* (affecting neighbouring loci), in *trans* (through RNA–protein or RNA–RNA interactions), and via ceRNA circuits with miRNAs [60, 61]. Despite this progress, non-model gymnosperms remain underexplored. Addressing this gap, we profile lncRNAs during early infection in *P. pinea* (resistant) and *P. radiata* (susceptible) challenged by *F. circinatum*, and concurrently survey pathogen-encoded lncRNAs during the pathogenicity. To our knowledge, this is the first identification and characterization of lncRNAs from *P. pinea* and *F. circinatum* within the PPC pathosystem, providing insights into the regulatory mechanisms engaged at the onset of disease.

Across published woody plant datasets, reported lncRNA catalog sizes vary with species, tissue breadth, and pipeline choices. The pine catalogs (8,783 lncRNAs in *P. radiata* and 5,255 in *P. pinea*) fall within the expected range for single-tissue experiments using a conservative multi-tool pipeline. For example, 3,689 lncRNAs were identified in *Paulownia tomentosa* under phytoplasma infection [62], 9,355 in *Populus* × *euramericana* infected by Melampsora [63], 6,417 in *Eucalyptus urophylla* under cold [64], and 3,094 in *Hevea brasiliensis* infected by *Colletotrichum gloeosporioides* [65]. In a comparable pathosystem, *P. radiata* infected by *F. circinatum* yielded 13,312 lncRNAs using a less conservative filtering pipeline [21]. Methodological non-standardization and the low primary-sequence conservation of lncRNAs (which limits homology-based discovery) make the results more variable [48]. It is also worth noting that, because host reads were aligned to the *P. taeda* reference genome, interspecific divergence may have influenced mapping efficiency and transcript reconstruction. Indeed, host mapping rates were consistently higher for *P. radiata* (~ 78–81%) than for *P. pinea* (~ 46–49%), which may have reduced sensitivity for transcript detection in the latter species.

The lncRNA catalogs generated here showed characteristic features consistently reported in plants and in other organisms [66–69]. These sequences were shorter in length, had fewer and shorter exons, with a dominance of intergenic lncRNAs (∼94.1% in *P. radiata* and 93.0% in *P. pinea*) and a smaller fraction of antisense and intronic classes. LncRNAs in *A. thaliana* generally show expression levels ~ 30–60-fold lower than mRNAs [70], and accordingly the lncRNAs we report here were expressed at relatively low levels in comparison with protein-coding genes. The GC content of the assembled transcripts of both pine species (45%) was similar to that of the transcriptome of other *Pinus* spp. such as *P. tecunumanii* (44%) [25]. Separately, the GC content in pine lncRNAs (41%) was lower than in protein-coding RNAs, a recurrent feature of plant lncRNAs attributed to distinct evolutionary pressures on ORFs [71]. Interestingly, lncNATs showed features that differed from the other lncRNA categories. This likely reflects their overlap with cognate protein-coding loci (in nearly 60% of loci), whereby biogenesis and expression are often coupled to the sense transcription unit, leading to distinct sequence/structure biases and dynamics [72]. For instance, at the FLC locus, the antisense lncRNA COOLAIR showed mutually exclusive sense–antisense transcription and contributed to cold-induced shutdown of FLC during vernalization [73]. Overall, the class proportions, genomic features, and expression patterns we observe are consistent with the architecture of lncRNAs reported across taxa, supporting the robustness of our identification pipeline.

LncRNAs are widely reported to contribute to the positive or negative regulation of gene expression [4]. One of the conserved mechanisms of action of the lncRNAs is their function as decoys by sequestering RNA-binding proteins (RBP), miRNAs or chromatin-modifying complexes [74]. It has been shown that some lncRNAs act as targets or target mimics of miRNAs, and that they regulate *Triticum aestivum* resistance to powdery mildew and stripe rust through miRNA-mediated pathways [75]. A subset of our pine lncRNAs overlaps hairpin-forming regions consistent with putative miRNA precursors, including MIR160, MIR1314, and MIR3701. Similar observations, where stress-responsive lncRNA loci either encode miRNA-like hairpins or participate in lncRNA–miRNA–mRNA (ceRNA) circuits, have been reported in woody systems under pathogen pressure [76, 77]. While we do not infer mature miRNA biogenesis without experimental validation, the co-occurrence of hairpin-bearing lncRNAs with defence-enriched coding neighborhoods supports post-transcriptional crosstalk in immunity [78]. Notably, we detected more lncRNA candidates with miRNA-like hairpins in *P. radiata* than in *P. pinea* (82 vs. 19). Family usage also differed (MIR160/MIR1314 in *P. radiata* vs. MIR160/MIR3701 in *P. pinea*), pointing to species-specific intersections with small-RNA pathways early after infection. MIR1314 appears conifer-restricted and has been linked to stress-related transcription factors in *Araucaria angustifolia*, suggesting roles at the hormone/abiotic–biotic interface [79], whereas MIR3701, more abundant in the resistant *P. pinea*, is a gymnosperm family connected to NBS-LRR/defence networks [80]. These patterns align with the earlier pathogen recognition reported for resistant pines in this pathosystem [20, 81].

Predicted lncRNA–mRNA pairs in our datasets are consistent with canonical roles of lncRNAs in chromatin modification, transcriptional regulation and posttranscriptional regulation [59]. *Fusarium circinatum* interaction targets were strongly enriched for transcriptional programs, including transcription by RNA polymerase II, regulation of transcription, RNA biosynthetic process or DNA-binding transcription factor activity. This pattern is consistent with lncRNAs interfacing with transcription factors and chromatin machinery to modulate gene expression. In *P. pinea*, the enrichment for translation initiation/translation factor activity terms is compatible with lncRNA effects on RNA-binding proteins (RBPs)-rich translation complexes. On the other hand, ADP/oxygen binding, malate metabolism/dehydrogenase activities, and ent-kaurene oxidation (GA biosynthesis) were enriched in lncRNA–mRNA pairs in *P. radiata*, regulatory roles also reported for lncRNAs in other plant species [82–84]. However, as noted above, the use of *P. taeda* as reference may affect inferred genomic distances and should be considered when interpreting proximity-based *cis* candidates.

We detected differentially expressed lncRNAs (DELncRNAs) during early infection in both pine species, with similar counts at 4 dpi (*P. radiata*: 37; *P. pinea*: 34), despite larger differences in coding DEGs reported for this pathosystems [20]. This scale is comparable to other biotic-stress datasets generated from single tissues and early time points. For example, 53 DELncRNAs in *P.* × *euramericana* leaves during early *Melampsora* infection [63], ~ 100–120 infection-related lncRNAs reported for *Paulownia* under phytoplasma challenge [85], and 15 lncNATs responsive to *F. oxysporum* infection in *A. thaliana* [7]. This supports that only a small fraction of lncRNAs is rapidly mobilized at early stages of pathogen perception. Many DELncRNAs identified here lie in *cis* proximity to defence-related coding genes. A subset al.so shows coordinated expression changes with nearby genes, which is consistent with local regulatory effects. In *P. radiata*, several DELncRNAs were located near perception/signaling and lignification nodes. In *P. pinea*, DELncRNA-*cis* pairs were comparatively enriched near redox and protein-homeostasis components. Although the *P. pinea cis* targets are consistent with a rapid attempt to stabilize cellular status and contain oxidative/proteotoxic stress after infection, the *P. radiata* configuration needs closer examination, as impaired pathogen perception leading to weaker downstream defence has been reported for this species [20, 81]. This suggests that, in *P. radiata*, the lncRNAs may indicate compensatory or delayed sensing rather than effective early recognition, a possibility that requires experimental testing. Consistent with a possible *cis* relationship in the lignin pathway, we detected PIRA.24844.1 (fold change + 6.47) adjacent to PITA_43179 (4CL3), and in Zamora-Ballesteros et al., (2022) the same lncRNA was detected (lncRNAPiRa.33098.2: fold change + 6.8) likewise adjacent to PITA_43179. This *cis*-regulation was predicted to be sense, downstream in the same strand, ~ 5,950-6,072 nt apart. Notably, in our dataset PITA_43179 did not pass DEG thresholds (fold change: +5.8; FDR: 0.09), indicating that *cis* coupling does not always produce a detectable mRNA change at this stage.

To complement *cis* evidence, we placed lncRNAs within co-expression modules to explore potential *trans*-associated candidates based on shared expression patterns. Importantly, because module-level GO enrichment and connectivity-based hub prioritization can be influenced by module size, these results should be interpreted with caution as hypothesis-generating rather than definitive evidence. Our co-expression analysis placed pine DELncRNAs into species-specific network contexts. In *P. radiata*, DELncRNAs occurred in several modules, none of which showed a significant eigengene-infection association. However, the module containing the largest number of DELncRNAs (ME4^PR^) was functionally coherent for defence chemistry and wall-adjacent processes (e.g., defence response, chitin binding, chalcone/phenylpropanoid terms, and naringenin-chalcone synthase activity). This suggests that, although infection-wide modulation of those modules was not detected, DELncRNAs are candidates positioned within defence-relevant co-expression neighborhoods in *P. radiata*. In contrast, *P. pinea* showed infection-responsive modules containing DELncRNAs with opposite signs of association. ME2^PP^ harbored most DELncRNAs and was positively associated with infection, with enrichment in plant immunity-related categories such response to oxidoreductase activity, flavonoid biosynthetic process, immune response and secondary metabolite biosynthetic process. Terms that have been associated with resistance to *C. gloeosporioides* of walnut fruit bracts [67]. In addition, ME5^PP^ was negatively associated with infection and was enriched for photosynthesis/chloroplast terms, consistent with a downshift of primary metabolism during infection. Thus, in *P. pinea*, modules containing DELncRNAs appear to shift with infection, whereas in *P. radiata* those DELncRNAs reside in defence-like modules without a significant infection-wide change. This may indicate that, although *P. radiata* possesses the defensive machinery, it is not properly activated, consistent with previous observations [20, 81]. Additionally, convergence on similar coding targets (e.g., carbohydrate flux via the raffinose pathway and redox buffering) occurs in distinct modules across hosts, suggesting species-specific *trans* use of lncRNAs to reach shared defence processes. This is compatible with the notion that plant lncRNAs show limited primary-sequence conservation while preserving positional or functional roles across species [86].

Across plants, lncRNA primary sequences show little conservation that further declines with evolutionary distance [4, 48]. Consistent with this, pine lncRNAs showed little similarity to angiosperm databases, and few sequence matches were detected between *P. pinea* and *P. radiata* despite shared tissue, age, and pathosystems. Notably, *P. pinea* displayed a higher apparent homology (39.1%) than *P. radiata* (22.7%). Given the limited cross-lineage similarity even for coding transcriptomes (pine vs. angiosperms) contrasted with high within-conifer xylem conservation (~ 78–82%; E-value ≤ 10⁻⁵) [87], we cannot draw robust conclusions regarding their sequence conservation. Positional or functional features can be conserved despite sequence divergence. For instance, conserved antisense transcript positions at FLC/COOLAIR-like loci across six grass species [88] and analogous DNA methylation-reader roles for human lncRNA UHRF1 protein-associated transcript (UPAT) and plant lncRNA AUXIN-REGULATED PROMOTER LOOP (APOLO) [89], may still share secondary structures [90]. However, the lack of chromosome-level assemblies for our study species precluded rigorous tests of positional or structural conservation.

Beyond the global transcriptional-control signal in *cis* and *trans*, our pathogen-side results highlighted ergosterol biosynthesis and cell-wall–degrading activities as plausible lncRNA-connected axes relevant to virulence in the pine–*F. circinatum* pathosystem. Host plants can sequester pathogen sterols through PR-1–type resistance proteins, thereby inhibiting pathogen growth as a resistance mechanism [91]. In our dataset, the sole down-regulated lncRNA in the pathogen during infection of *P. pinea* (vs. *P. radiata*) was *cis*-positioned near sterol-biosynthetic loci. Consistently, genes in this pathway, including δ14-sterol reductase (*ERG24*), were expressed at lower levels when *F. circinatum* infected resistant hosts [18]. Our results extend this hypothesis by suggesting that sterol-pathway genes and nearby fungal lncRNAs (FC072V.9742.1) show host-dependent expression patterns that may be sensitive to host resistance. In parallel, during infection of *P. pinea* we observe up-regulation of lncRNAs potentially *cis*-regulating genes associated with the cell-wall degradation, consistent with a reinforced offensive program under a more restrictive host environment. This aligns with previous analysis showing early induction of secreted enzymes/CWDEs in *P. pinea* upon *F. circinatum* infection [20]. Taken together, these data support fungal lncRNAs as candidates potentially modulating virulence-associated programs under different host defenses.

In summary, this work contributes to expanding the molecular framework of the *Pinus–Fusarium circinatum* interaction by providing evidence for a new regulatory layer mediated by long non-coding RNAs. By jointly profiling host and pathogen transcriptomes, we report species-specific lncRNA responses and network architectures that may underlie resistance and virulence strategies. At the lncRNA level, the resistant *P. pinea* showed a more coordinated infection-associated regulatory landscape, with DELncRNAs enriched in infection-responsive programs that couple defence activation with down-regulation of photosynthesis/chloroplast functions. By contrast, in susceptible *P. radiata*, DELncRNAs were embedded in defence-related co-expression neighborhoods but without a strong infection-wide module shift, consistent with a diffuse or incompletely engaged regulatory response at 4 dpi. Beyond providing the first catalogues of *P. pinea* and *F. circinatum* lncRNAs, our study underscores the importance of integrating non-coding RNA regulation into forest pathosystem research. Importantly, our conclusions are based on computational predictions from this dataset. Hub lncRNAs/genes highlighted here should be viewed as priority candidates that will require independent validation to confirm their roles and improve confidence in their regulatory relevance. These findings open avenues for exploiting lncRNA-based mechanisms to enhance conifer resilience against emerging diseases.

## Supplementary Information

Supplementary Material 1.

Supplementary Material 2.

## Data Availability

The datasets supporting the conclusions of this article are included within the article and its additional files. Raw sequencing reads are deposited in the NCBI Sequence Read Archive under BioProject PRJNA702546 (runs SRR13737940–SRR13737953) and also in Zenodo (DOI: 10.5281/zenodo.17591267). The analysis code (scripts and documentation) is available on GitHub and archived in Zenodo: doi:10.5281/zenodo.17591556. Fasta files with lncRNAs identified in this work are included in the same Zenodo record.

## Abbreviations

ADSS: Adenylosuccinate synthetase
BGLG: Beta-glucosidase G
BLAST: Basic Local Alignment Search Tool
bp: Base pair
ceRNA: Competing endogenous RNA
CNCI: Coding-Non-Coding Index
CPAT: Coding-Potential Assessment Tool
CPC2: Coding Potential Calculator 2
CTF1-BETA: Cutinase Transcription Factor 1 Beta
CWDE: Cell wall–degrading enzyme
DEG: Differentially expressed gene
DELncRNA: Differentially expressed long non-coding RNA
dpi: Days post inoculation
ET: Ethylene
FDR: False discovery rate
FEELnc: Fast and Efficient Extraction of Long Non-Coding RNAs
FPKM: Fragments per kilobase of exon per million mapped reads
GA: Gibberellin
GFF/GTF: General Feature Format / Gene Transfer Format
GO: Gene Ontology
JA: Jasmonic acid
kb: Kilobase
lncRNA: Long non-coding RNA
lncNAT: Natural antisense long non-coding RNA
lincRNA: Long intergenic non-coding RNA
mRNA: Messenger RNA
ncRNA: Non-coding RNA
NTRA: Thioredoxin reductase
ORF: Open reading frame
PAL: Phenylalanine ammonia-lyase
PCA: Principal component analysis
PCR: Polymerase chain reaction
PLEK: Predictor of Long non-coding RNAs and mRNAs based on k-mer scheme
PR1: Pathogenesis-related protein 1
PRR: Pattern recognition receptor
RNA-seq: RNA sequencing
rRNA: Ribosomal RNA
SA: Salicylic acid
SAM/BAM: Sequence Alignment/Map / Binary Alignment/Map
SRA: Sequence Read Archive
SVM: Support vector machine
TF: Transcription factor
TIR: Toll/interleukin-1 receptor homology domain
TOM: Topological Overlap Matrix
TCA: Tricarboxylic acid
tRNA: Transfer RNA
UTR: Untranslated region
VST: Variance-stabilizing transformation
WGCNA: Weighted Gene Co-Expression Network Analysis
