# Supplementary material for: Dual RNA-seq analysis reveals differences in defensive lncRNA expression in Pinus spp. with varying susceptibility to Fusarium circinatum
Source: BMC Plant Biol. 2026 Jan 23;26:326. doi: 10.1186/s12870-026-08182-w (PMC12911216; doi:10.1186/s12870-026-08182-w)
Supplement: Supplementary file 1 — Supplementary Material 1. [file 12870_2026_8182_MOESM1_ESM.docx]

Additional Figures


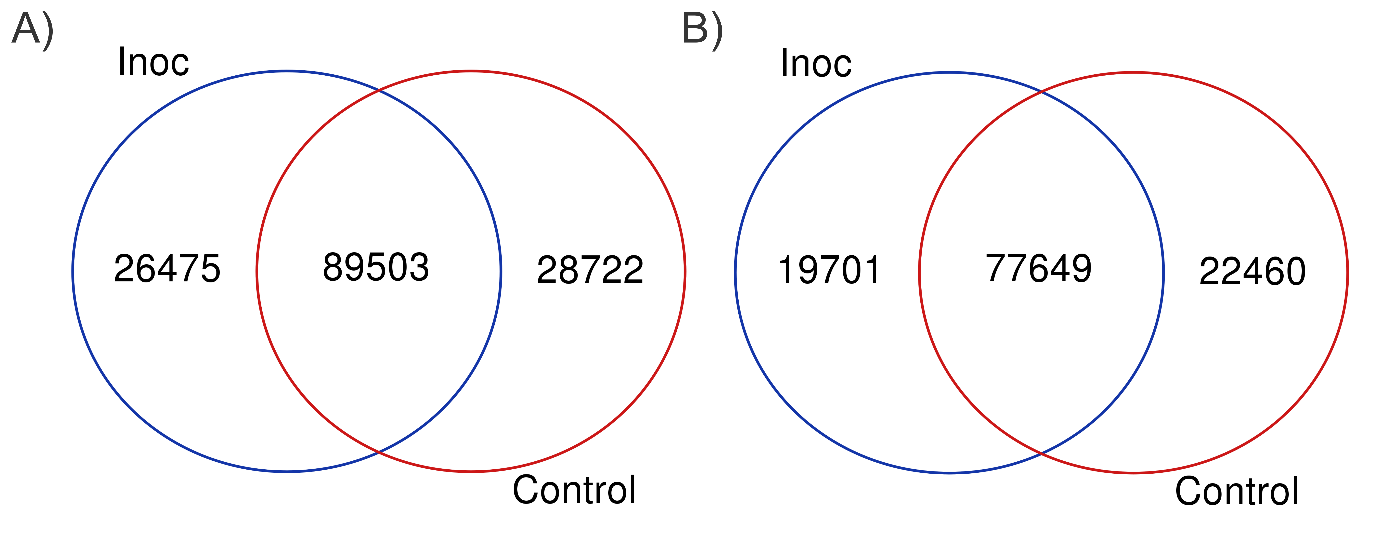


**Figure S1**. Venn diagram showing the number of assembled transcripts in inoculated and control transcriptomes of *P. radiata* (A) and *P. pinea* (B).


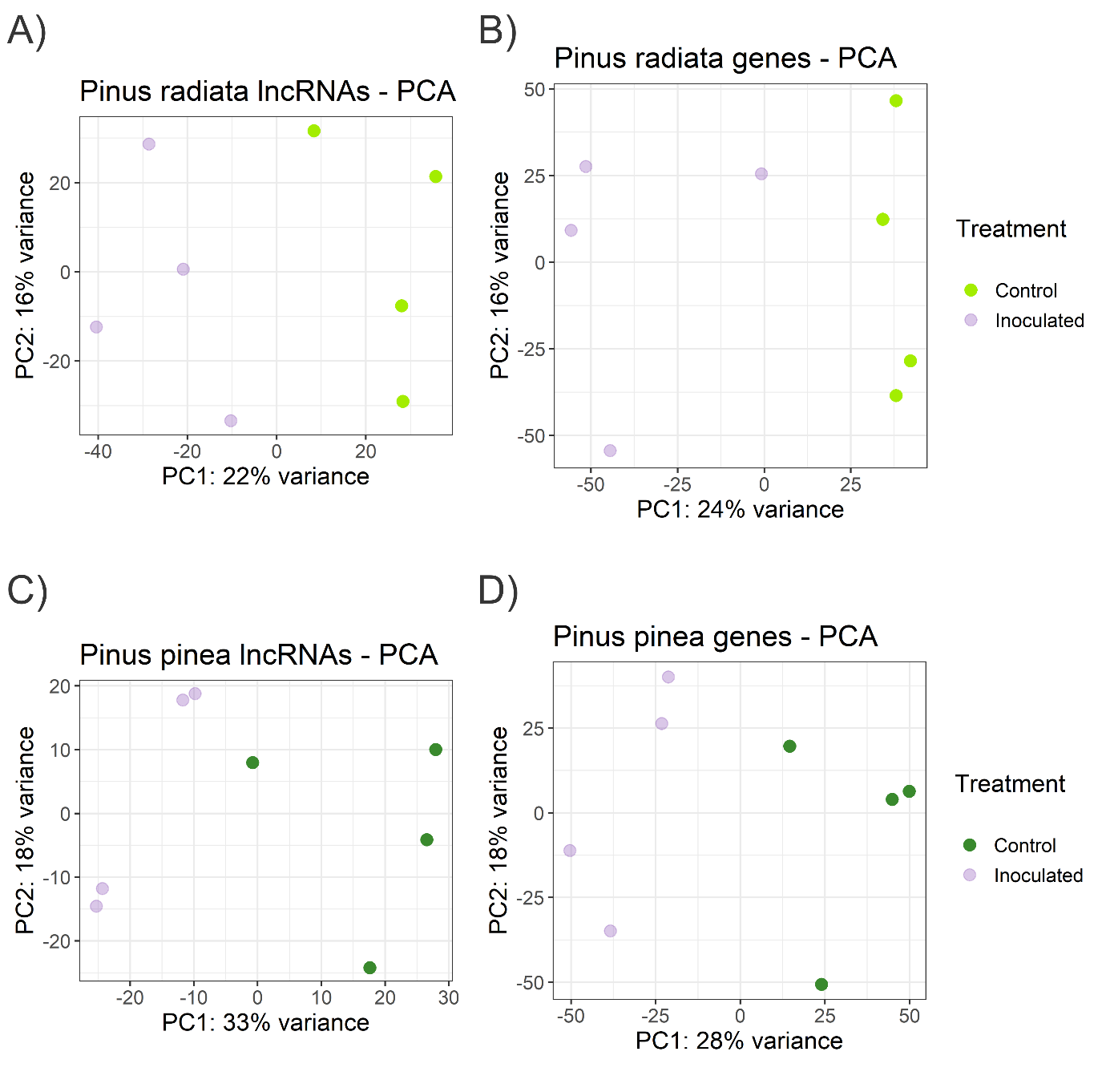


**Figure S2**. Two-dimensional scatterplot of the principal component analyses (PCA) for *Pinus radiata* (A) lncRNAs and (B) protein-coding RNA, and *P. pinea* (C) lncRNAs and (D) protein-coding RNA, based on rlog-transformed counts.


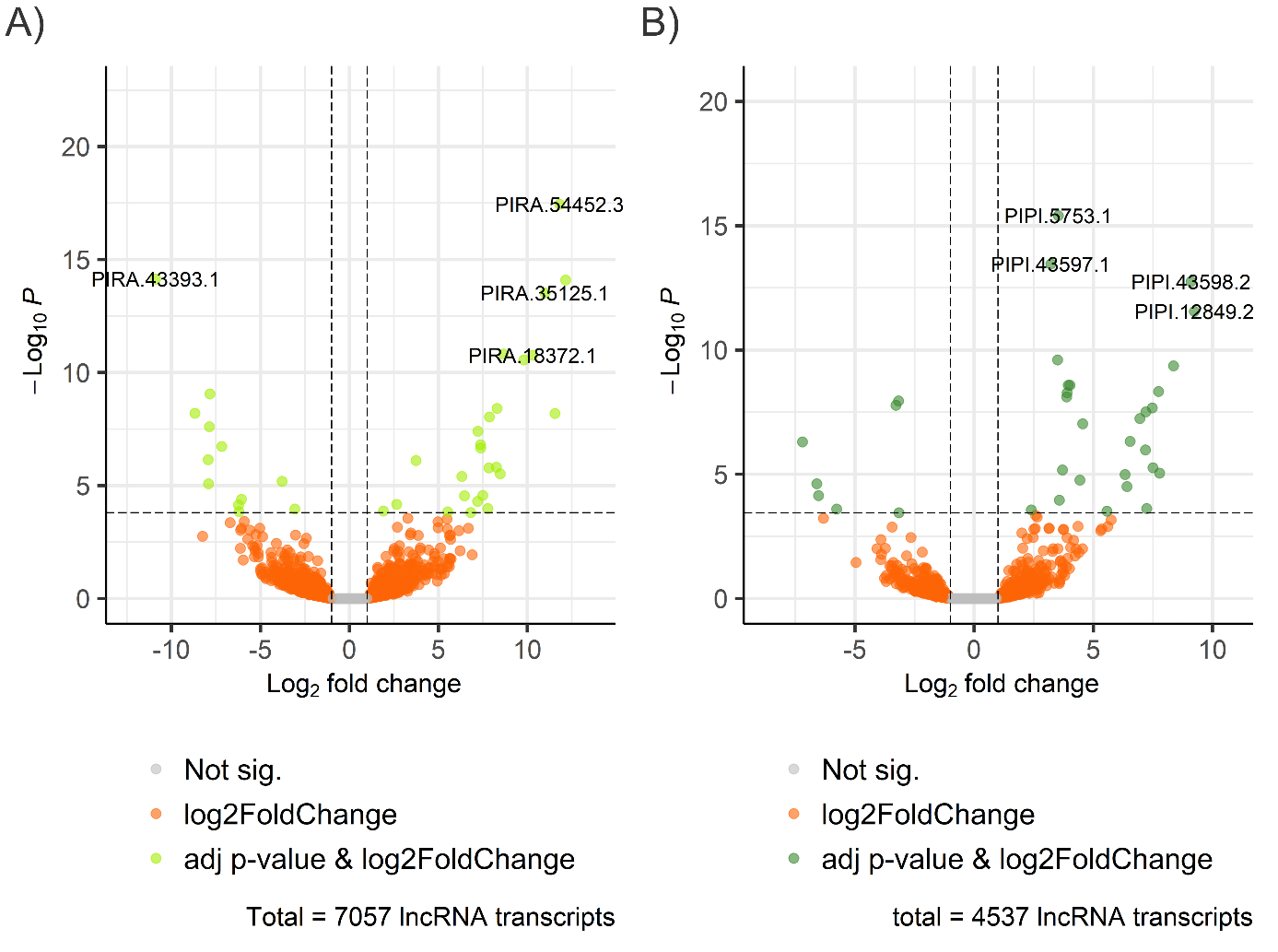


**Figure S3**. Volcano plots representing the differentially expressed lncRNAs (DELncRNAs) in (**A**) *Pinus radiata* and (**B**) *P. pinea* infected by *Fusarium circinatum*. The x-axis represents log_2_ fold change, and the y-axis represents log_10_ p-value. P-value < 0.05 and |log_2_ fold change| ≥ 1.


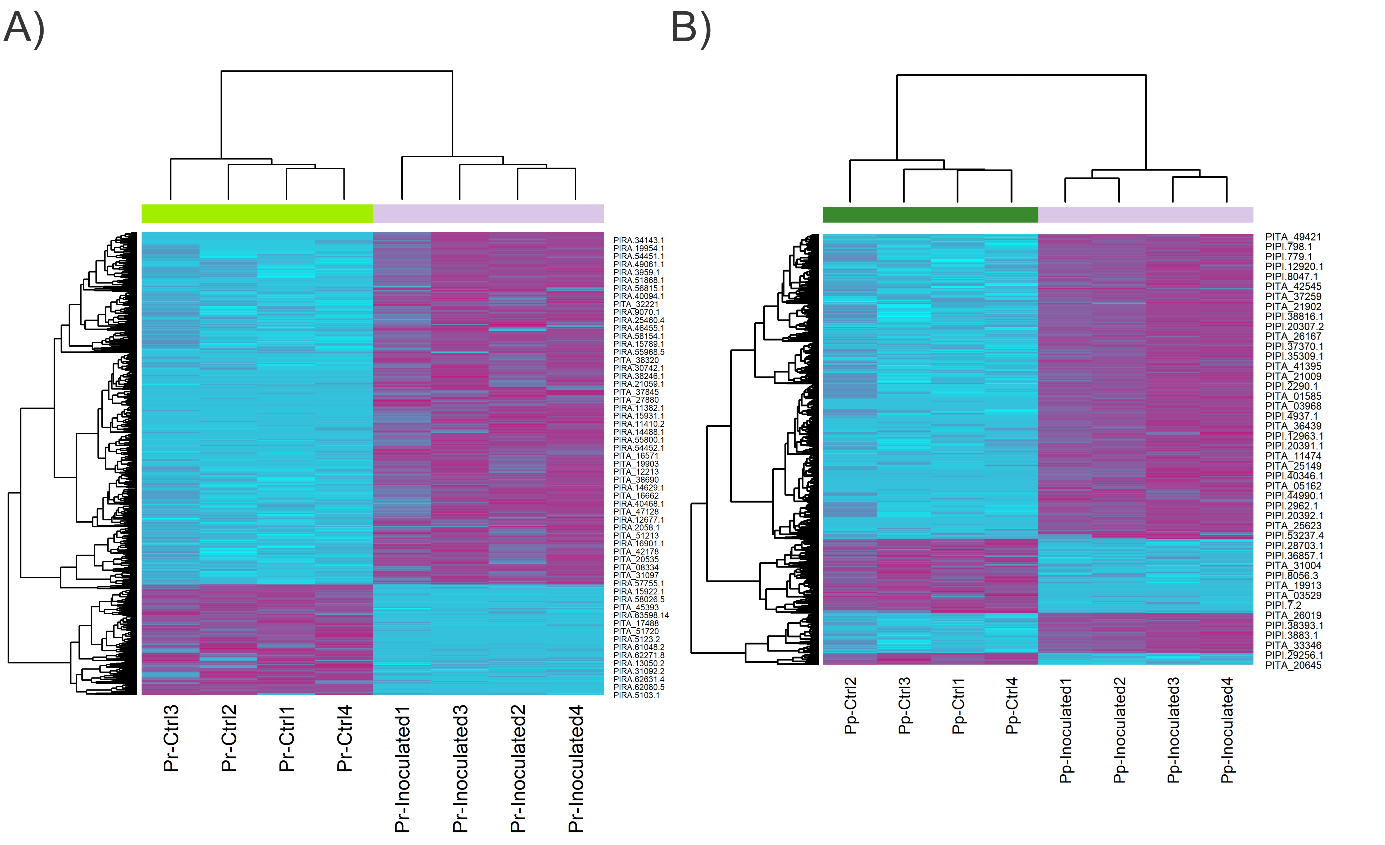


**Figure S4**. Hierarchical clustering plot of the differentially expressed genes of (A) *Pinus radiata* and (B) *Pinus pinea* in response to *F. circinatum*. The plots show the scaled expression levels of the genes. Different columns represent different libraries, and different rows represent the differentially expressed genes. Purplish: relatively high expression; Blue: relatively low expression.


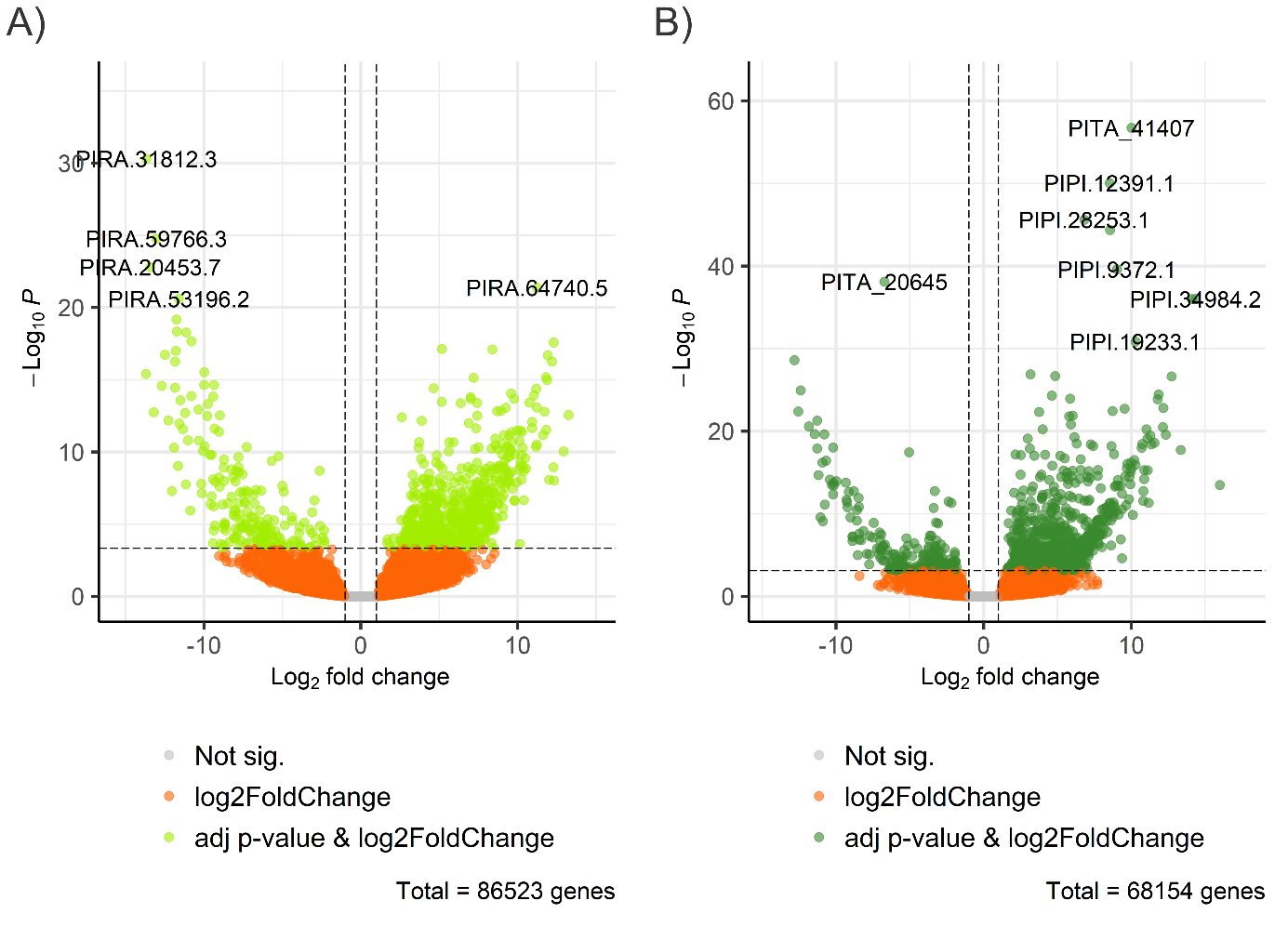


**Figure S5**. Volcano plot representing the differentially expressed genes (DEGs) in (A) *Pinus radiata* and (B) *Pinus pinea* infected by *Fusarium circinatum*. The x-axis represents log2 fold change, and the y-axis represents log_10_ p-value. Adjusted p-value < 0.05 and |log2 fold change| ≥ 1.


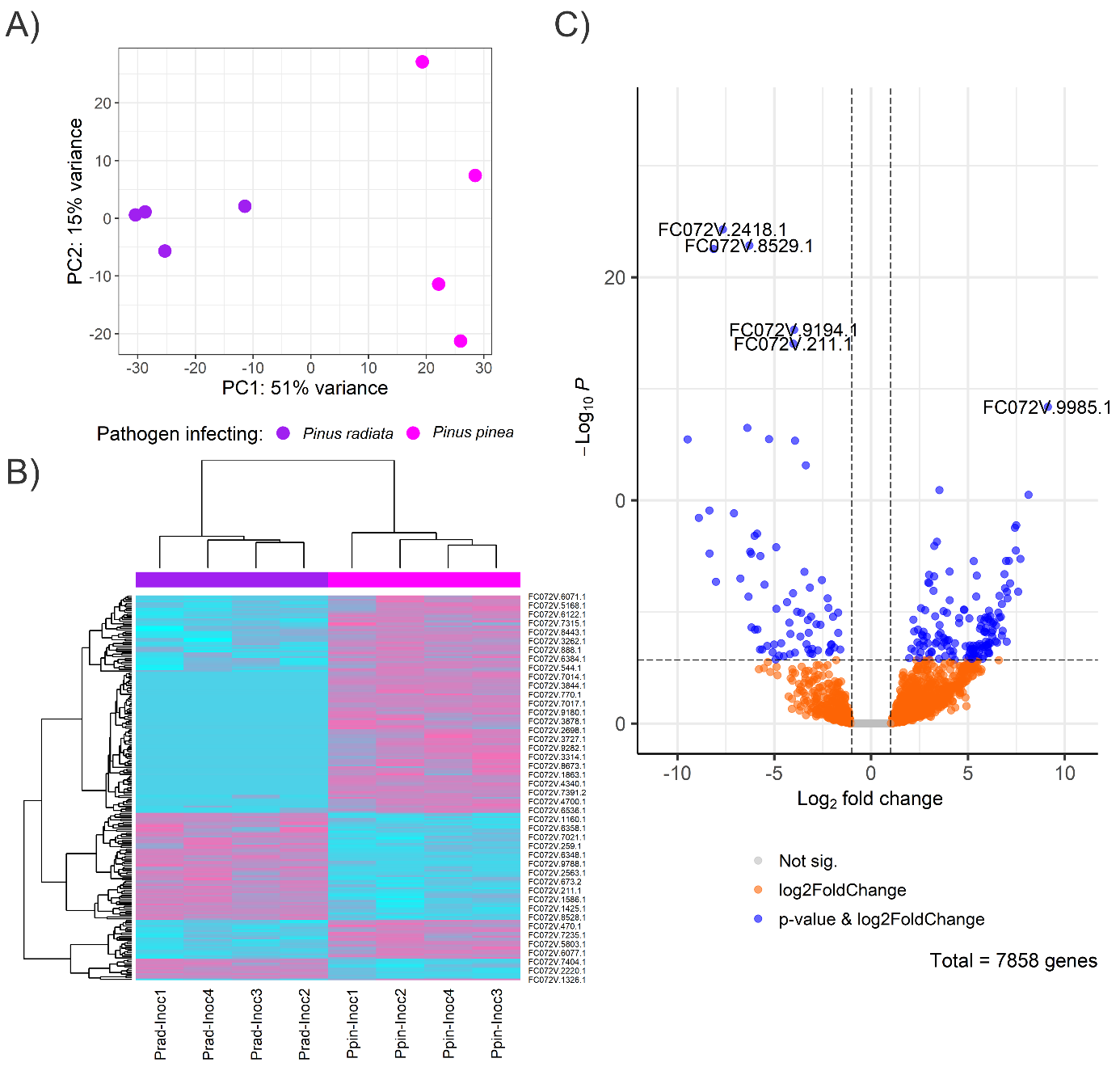


**Figure S6**. (A) Two-dimensional scatterplot of the principal component analyses (PCA) for *Fusarium circinatum* protein-coding transcripts under *Pinus pinea* infection compared to *P. radiata* infection based on rlog-transformed counts. (B) Hierarchical clustering plot of the differentially expressed genes of *F. circinatum* under *P. pinea* infection compared to *P. radiata* infection. The plots show the scaled expression levels of the genes. Different columns represent different libraries, and different rows represent the differentially expressed genes. Pinkish: relatively high expression; Blue: relatively low expression. (C) Volcano plot representing the differentially expressed genes (DEGs) in *F. circinatum* under *P. pinea* infection compared to *P. radiata* infection. The x-axis represents log2 fold change, and the y-axis represents log_10_ p-value. Adjusted p-value < 0.05 and |log2 fold change| > 1.


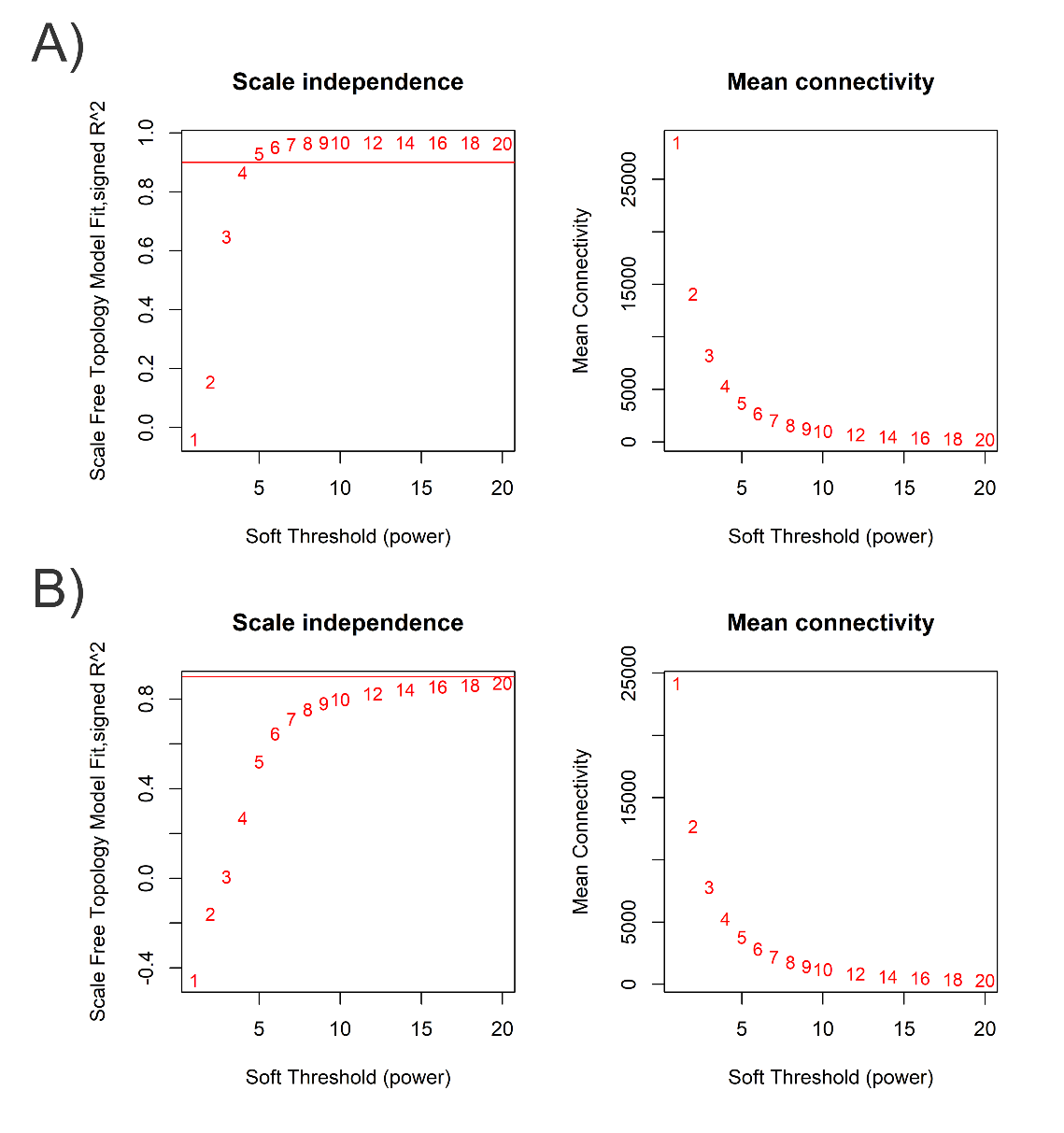


**Figure S7**. Selection of soft-thresholding power (β) for network construction. A) For *P. radiata*, a power of β = 6 was selected, yielding a scale-free topology fit of R² = 0.952, slope = −1.88, and mean connectivity = 2,680. B) For *P. pinea*, a power of β = 12 was chosen, corresponding to R² = 0.824, slope = −1.39, and mean connectivity = 852.


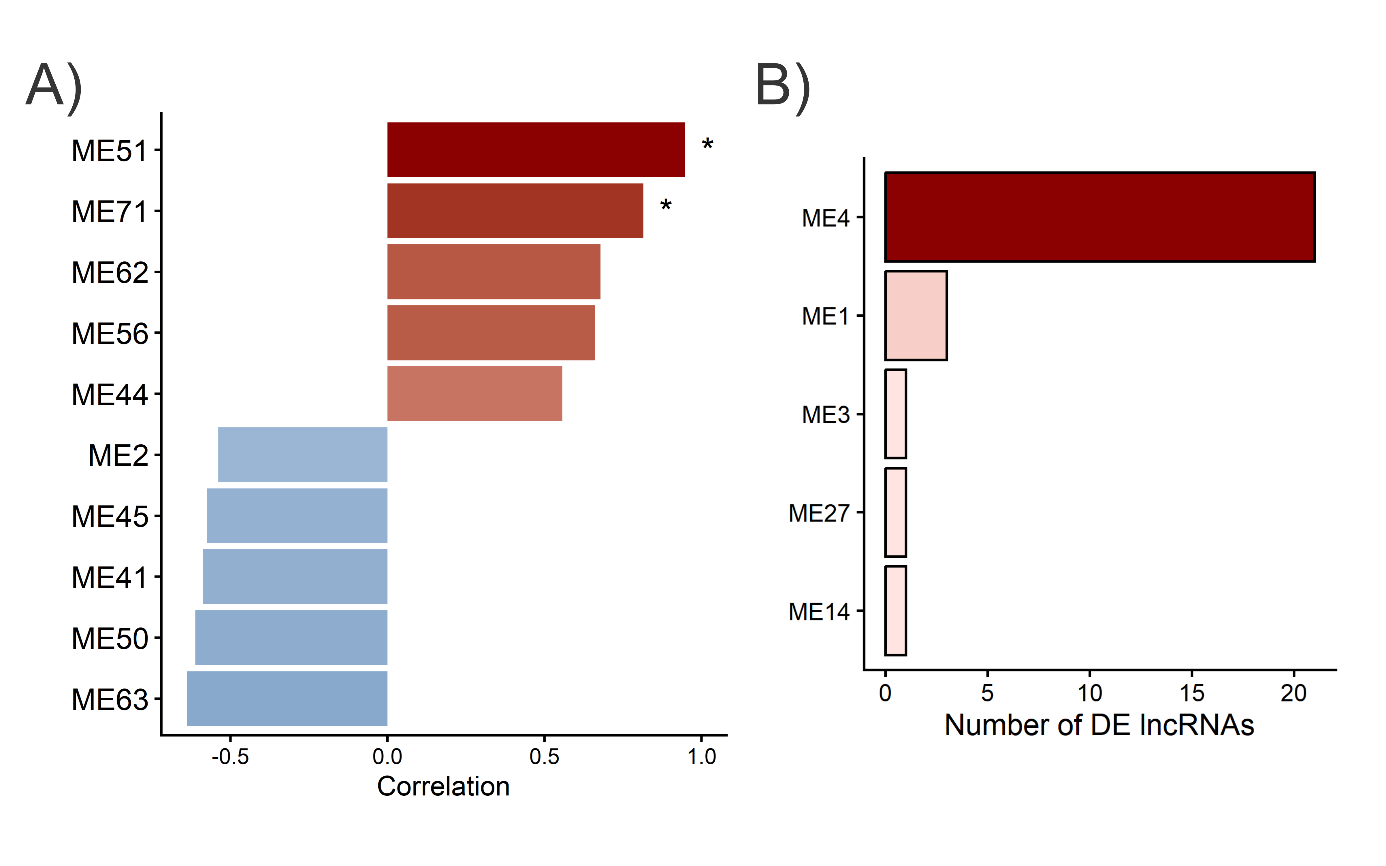


**Figure S8.** Co-expression modules and network connectivity of DELncRNAs in *P. radiata*. A) Top 10 modules ranked by absolute correlation with infection condition. Bars represent correlation coefficients; asterisks indicate significant associations (|r| > 0.6, p < 0.05). B) Distribution of differentially expressed lncRNAs (DELncRNAs) across the five modules in which they were detected.


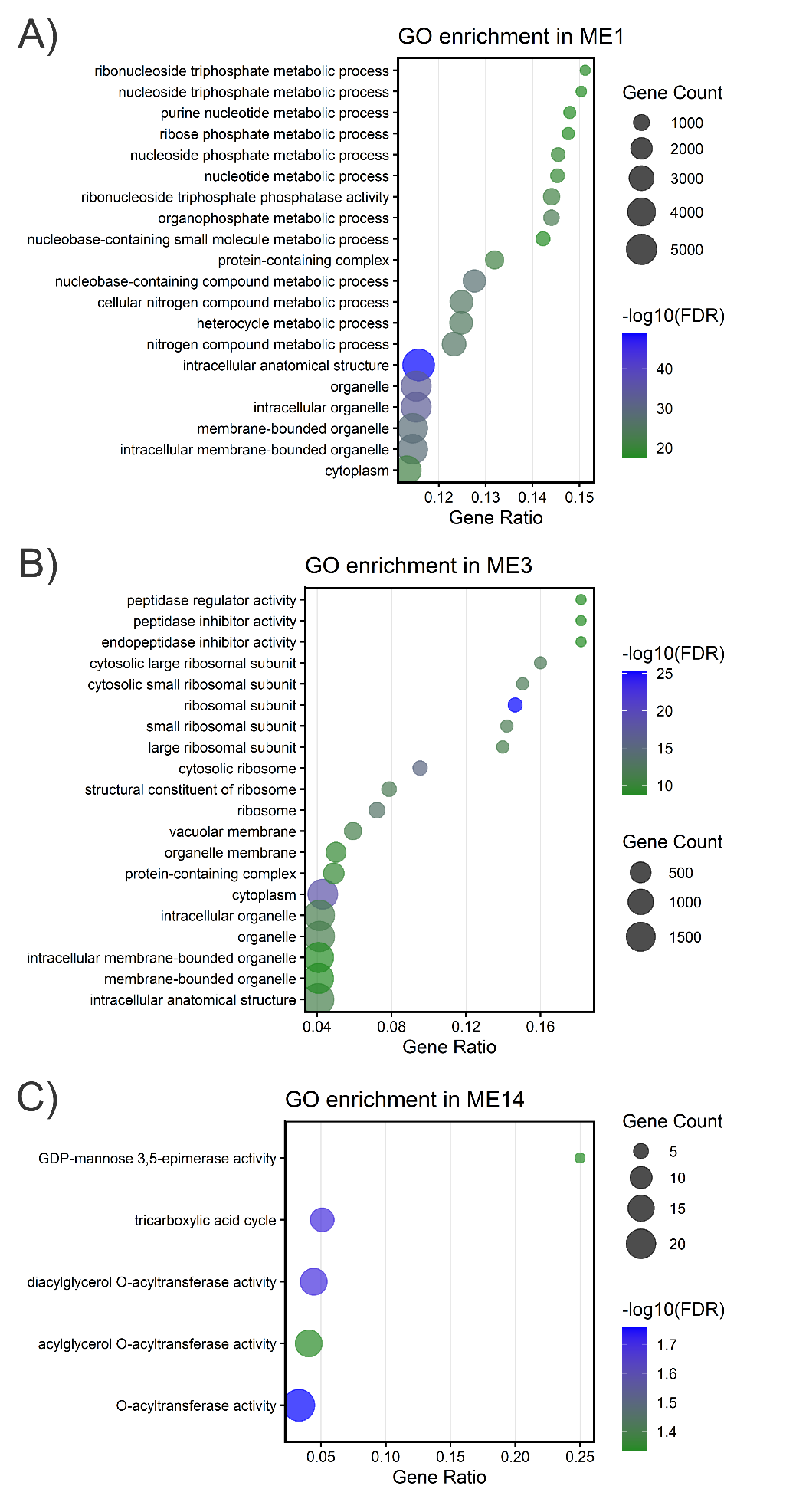


**Figure S9**. Top enriched Gene Ontology (GO) terms associated with genes from modules (A) ME1, (B) ME3 and (C) ME14, harboring DELncRNAs in *P. radiata*. No GO enriched terms were found in ME27.


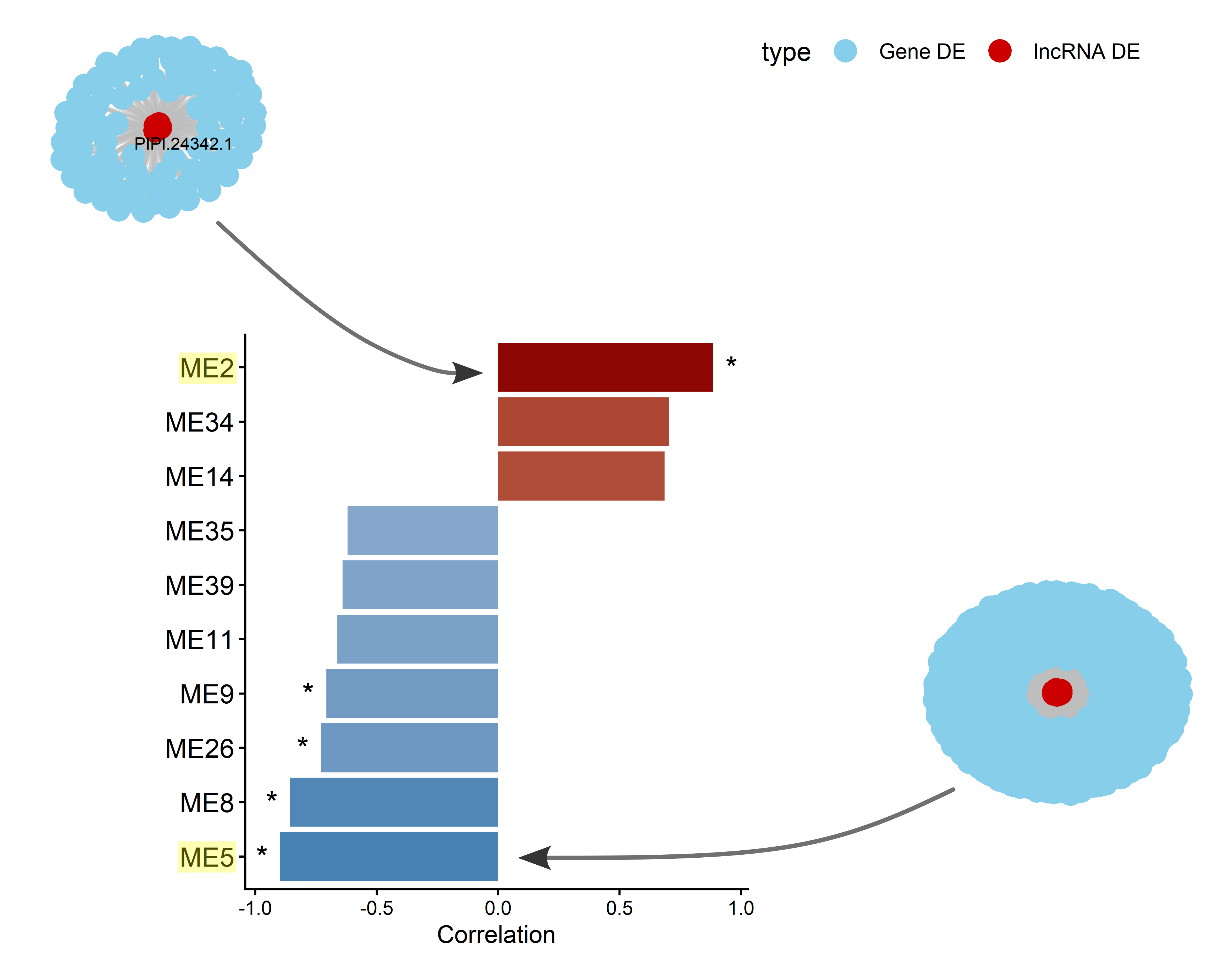


**Figure S10.** Top 10 co-expression modules ranked by correlation with infection condition in *P. pinea*. Bars represent correlation coefficients; asterisks indicate significant associations (|r| > 0.6, p < 0.05). The global co-expression network of the modules with DELncRNAs that, in turn, showed the strongest correlation with the infection is represented. The yellow-highlighted modules contain DELncRNAs. The network was constructed from DELncRNAs and differentially expressed (DE) genes of common modules. Red nodes represent DELncRNAs and blue nodes represent DEGs.


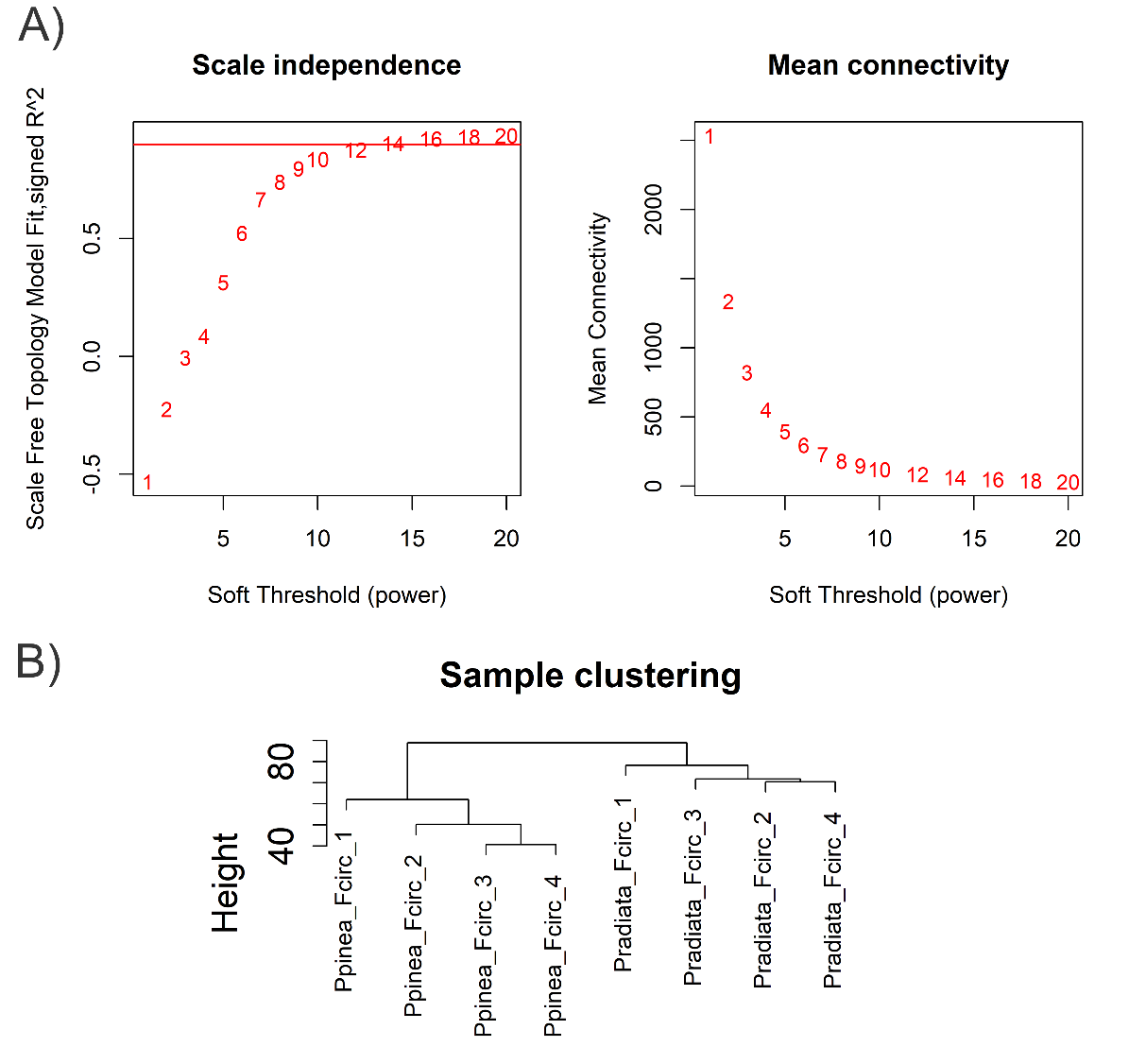


**Figure S11**. A) Analysis of the scale-free topology criterion and mean connectivity across different soft-thresholding powers. A threshold of β = 12 was selected (horizontal red line indicates the standard R² = 0.90 cut-off). B) Hierarchical clustering of samples based on variance-stabilized expression values.
